# Myb Transcription Factors and Light Regulate Sporulation in the Oomycete *Phytophthora infestans*

**DOI:** 10.1371/journal.pone.0092086

**Published:** 2014-04-04

**Authors:** Qijun Xiang, Howard S. Judelson

**Affiliations:** Department of Plant Pathology and Microbiology, University of California Riverside, Riverside, California, United States of America; University of Wisconsin - Madison, United States of America

## Abstract

Life cycle progression in eukaryotic microbes is often influenced by environment. In the oomycete *Phytophthora infestans*, which causes late blight on potato and tomato, sporangia have been reported to form mostly at night. By growing *P. infestans* under different light regimes at constant temperature and humidity, we show that light contributes to the natural pattern of sporulation by delaying sporulation until the following dark period. However, illumination does not permanently block sporulation or strongly affect the total number of sporangia that ultimately form. Based on measurements of sporulation-induced genes such as those encoding protein kinase Pks1 and Myb transcription factors Myb2R1 and Myb2R3, it appears that most spore-associated transcripts start to rise four to eight hours before sporangia appear. Their mRNA levels oscillate with the light/dark cycle and increase with the amount of sporangia. An exception to this pattern of expression is *Myb2R4*, which is induced several hours before the other genes and declines after cultures start to sporulate. Transformants over-expressing *Myb2R4* produce twice the number of sporangia and ten-fold higher levels of *Myb2R1* mRNA than wild-type, and chromatin immunoprecipitation showed that Myb2R4 binds the *Myb2R1* promoter *in vivo*. *Myb2R4* thus appears to be an early regulator of sporulation. We attempted to silence eight Myb genes by DNA-directed RNAi, but succeeded only with Myb2R3, which resulted in suppressed sporulation. Ectopic expression studies of seven Myb genes revealed that over-expression frequently impaired vegetative growth, and in the case of *Myb3R6* interfered with sporangia dormancy. We observed that the degree of silencing induced by a hairpin construct was correlated with its copy number, and ectopic expression was often unstable due to epigenetic silencing and transgene excision.

## Introduction

Eukaryotic microbes typically occupy environments in which fluctuating extrinsic factors such as temperature, light, humidity, and nutrients influence progression through the life cycle. Reactions to these conditions are mediated by transcription factors (TFs), protein kinases, G protein-coupled receptors, and other sensors, transducers, and effectors [1], [2]. Many environmental responses have evolved to enhance fitness. For example, sporulation at high humidity is common in fungi that make spores that must imbibe water to germinate [3], [4]. Often sporulation is suppressed in low oxygen environments which may not favor aerial dissemination [5], [6]. In some fungi, solar radiation triggers synthesis of UV-protectants such as carotenoids, or inhibits production of spores that lose viability in light [7]. Other fungi and some slime molds sporulate more intensely in light, perhaps since this signals their presence on the surface of a growth substrate, which may aid spore dispersal [8], [9].

How life cycles are integrated with environmental cues is now understood in many fungi, particularly in regards to light [1]. In *Aspergillus nidulans*, for example, light perception by phytochromes induces Bristle (brlA) protein, a TF that regulates conidiation [10], [11]. In *Neurospora crassa*, the activity of WC (white collar) TFs are regulated by a light-sensing chromophore within the WC protein [7]. Other light-sensing proteins include the cryptochrome/photolyase, opsin, and vivid families [1], [12]. Each protein family tends to respond to different wavelengths of light, leading to distinct cellular responses.

Frequently occupying similar niches as fungi, and also sensitive to environment, are oomycetes, which are eukaryotic microbes in the Kingdom Stramenopila. A major genus is *Phytophthora*, which includes many important plant pathogens [13]. These grow by ramifying hyphae within the host and then form sporangia on plant surfaces, which later germinate by either developing zoospores or directly extending germ tubes. High humidity, which is common at night, is necessary for sporulation in most species of *Phytophthora* and relatives such as downy mildews [14]–[16]. Light may also influence sporulation, although data are contradictory with both inhibitory and enhancing effects being reported (rev. in [17]). Sporangia germination is temperature-sensitive, with cool conditions favoring zoospore release over the direct germination [18]. Chemical signals influence zoospore swimming, encystment, and the directional growth of germ tubes [19]. A few genes involved in sporulation have been characterized [20], [21], but it is unknown whether their expression or activity is regulated by environmental factors, including light.

In this study, we examine the interplay between light and regulators such as Myb TFs in the sporulation of *P. infestans*, the species responsible for late blight of potato and tomato. Light is shown to suppress sporulation on plants and artificial media, and influence the transcription of sporulation-associated genes including TFs in the Myb family. In an earlier report, we identified 16 R2R3 and R1R2R3-type Myb domain TFs from *P. infestans*, and showed that eight are up-regulated during sporulation [22]. Here, the use of light to synchronize sporulation helped to reveal that one Myb gene *(Myb2R4)* is transcribed earlier than other genes induced during spore development. Chromatin immunoprecipitation and ectopic expression studies supported the role of Myb2R4 as a regulator of sporulation. Ectopic expression and gene silencing also yielded insight into the activities of other Myb TFs, and technical factors that influence transgene expression and gene silencing.

## Materials and Methods

### Growth and development of *P. infestans*

Most experiments involved isolate 1306, an A1 mating type strain from tomato in California, USA. Cultures were maintained at 18°C on rye-sucrose agar in the dark, and developmental stages isolated as described [23]. In brief, sporangia were extracted from cultures by adding water and rubbing with a glass rod. To obtain nonsporulating mycelia, sporangia were inoculated into clarified broth and hyphae harvested after 72 hr. Zoosporogenesis was initiated by placing sporangia in 10°C water for 30 min, with zoospores being released after an additional 90 min of incubation. Cysts were obtained by adding 0.25 mM CaCl_2_ to zoospores and vortexing for 1 min. Germinated cysts were made by incubating cysts for 6 hr in water at 18°C. Directly germinated sporangia were obtained by placing sporangia for 4 hr in clarified rye-sucrose broth at 18°C. Some studies also involved isolate E13a, an A2 mating type strain from potato in Egypt. Student's t-test was used to assess differences in growth, sporulation, or germination between treatments or strains.

For studies of sporulation with different light regimes, plates were stored upside down in sealed transparent polystyrene containers containing water-saturated towels, in a Percival I-36LLVL incubator outfitted with cool-white fluorescent lighting that yielded 95 µmoles/m^2^/s at the culture surface. A digital thermometer and hygrometer (National Institute of Standards and Technology traceable) indicated that temperature and humidity were constant at 18°C and 98%, respectively. Day/night cycles were 12 hr each; to maintain 18°C within cultures during illumination, it was necessary to set the temperature control of the incubator to 17°C. Cultures exposed to continuous darkness were wrapped in light-tight black bags in the same incubator. Sporangia were washed from plates in water and counted. To increase contrast in photographic images, some experiments included a mixture of blue and red food coloring (FD&C red #40 and FD&C blue #1, respectively) in the agar medium.

Plant infection assays used tomato leaflets from five-week plants of cultivar New Yorker. Inoculations employed zoospores at 5×10^4^ per ml, which were applied using a sprayer to each leaflet. These rested on 1.5% water agar plates in sealed plastic bags containing water-saturated towels. A digital thermometer and hygrometer indicated that temperature and humidity were constant, using the conditions noted above. Spore counts were normalized to leaf surface area, which was measured using ImageJ.

### Gene sequences and nomenclature

Sequences were obtained from the *P. infestans* database maintained by the Broad Institute of MIT and Harvard. In our prior work [22], most of the Myb genes were classified as “Myb2” or “Myb3” types. These encode proteins containing two Myb repeats corresponding to the R2R3 domains of human c-Myb, or three repeats corresponding to the R1R2R3 domains of human c-Myb, respectively. For this study, some gene models were corrected and these are presented in Table S1.

### Polymerase chain reaction (PCR)

For reverse transcription-PCR (RT-PCR), RNA was extracted using the RNeasy Plant Mini kit (Qiagen) from tissues ground under liquid nitrogen, treated with RQ1 DNAse (Promega), and cDNA synthesized using the Maxima RT-PCR kit (Thermo). PCR was then performed using the primers shown in Table S2. All primers were predicted to be gene-specific, except for the CRN2 primers which detected a small family. For quantitative PCR (qPCR), primers were tested using a dilution series of template and accepted if efficiencies were above 94%. Amplifications were performed using the Dynamo SYBR Green kit (Thermo) with the following program: 95°C for 15 min, followed by 40 cycles of 94°C for 30 sec, 55°C to 60°C (depending on primer) for 30 sec, and 72°C for 30 sec. Melt curves were generated at the end of each run to test the fidelity of amplification. Expression levels were calculated using the ΔΔC_T_ method, using a constitutive gene (ribosomal protein S3a, PITG_11766) as a control [24], [25]. Values are from two biological replicates, with three technical replicates each. qPCR was also used to determine transgene copy number, using DNA extracted as described [26].

### Transgenic *P. infestans*

Transformations used the protoplast method [27]. Silencing constructs were based in pTOR (Genbank accession EU257520), in which the *ham34* promoter was used to drive transcription of a cassette containing about 400 nt of sense sequences, the *Ste20* intron, and antisense sequences [28], [29]. pTOR also contains the *nptII* gene for selection using G418. Transformants were tested for silencing using semiquantitative RT-PCR, and then RT-qPCR, using the primers shown in Table S2. Over-expression constructs were also based in pTOR, using the relevant gene amplified from cDNA using a C-terminal primer that incorporated a FLAG (DYKDDDDK) tag. Purification of transformants typically involved generating and encysting zoospores (which are usually mononucleate; [30]), plating the cysts on 2% water agar, and transferring single cysts to new rye-sucrose plates lacking G418 using a fine needle. Expression of FLAG-tagged protein was detected by western blot analysis of denaturing polyacrylamide gels using anti-FLAG primary and horseradish peroxidase-conjugated secondary antibodies (Sigma), with detection using chemoluminescence (ECL kit, GE Healthcare). RT-qPCR was used to compare expression levels in transformants and wild-type.

### Chromatin immunoprecipitation

The method was adapted from Strahl-Bolsinger et al. [31]. Hyphae in 36 ml stationary liquid cultures were mixed with formaldehyde to a final concentration of 1%, incubated for 15 min using a shaker at 50 rpm, and then for another 5 min with 1.8 ml of 2.5 M glycine. Hyphae were recovered by filtration, washed twice with 20 ml phosphate-buffered saline (PBS), frozen in liquid nitrogen, and ground with 0.4 mm glass beads. Samples were suspended in 0.6 ml of lysis buffer (50 mM HEPES 7.5, 140 mM NaCl, 1 mM EDTA, 1% Triton X-100, 0.1% sodium deoxycholate, 1 mM PMSF), sonicated four times for 30 sec at power setting 40 of a Fisher Sonic Dismembrator 550, and clarified by centrifugation at 4650× *g* at 4°C for 5 min. Immunoprecipitations used either anti-FLAG antibody or control IgG (Sigma). Prior to being added to the chromatin, 2 µg of antibody was mixed with 10 µl of Protein G magnetic beads (Sigma) in PBS for 1 hr at room temperature, washed three times in the same buffer, and resuspended in 50 µl of the same. The antibody/bead/chromatin mixture was incubated overnight at 4°C, and then bead-bound material was recovered using a magnet, washed twice in 0.6 ml lysis buffer, once in 0.6 ml of wash buffer (0.1 M Tris 8.0, 0.25 M LiCl, 0.5% sodium deoxycholate, 1 mM EDTA, 1 mM PMSF, 1 µl of protease inhibitor mix from Sigma), and twice in TE (10 mM Tris-1 mM EDTA pH 8.0). Beads were then resuspended in 0.2 ml TE containing 1% sodium dodecyl sulfate and held at 65°C overnight to reverse cross-links. After adding NaCl to 0.15 M, samples were incubated at 42°C for 2 hr with proteinase K (Sigma) at 1 mg/ml, extracted once with 1∶1 phenol∶chloroform, precipitated with ethanol, and resuspended in 100 µl water. A fraction was resolved by electrophoresis to check the size of the DNA, and 6 µl aliquots were used for qPCR.

## Results

### Light influences sporulation timing

**Figure 1:**
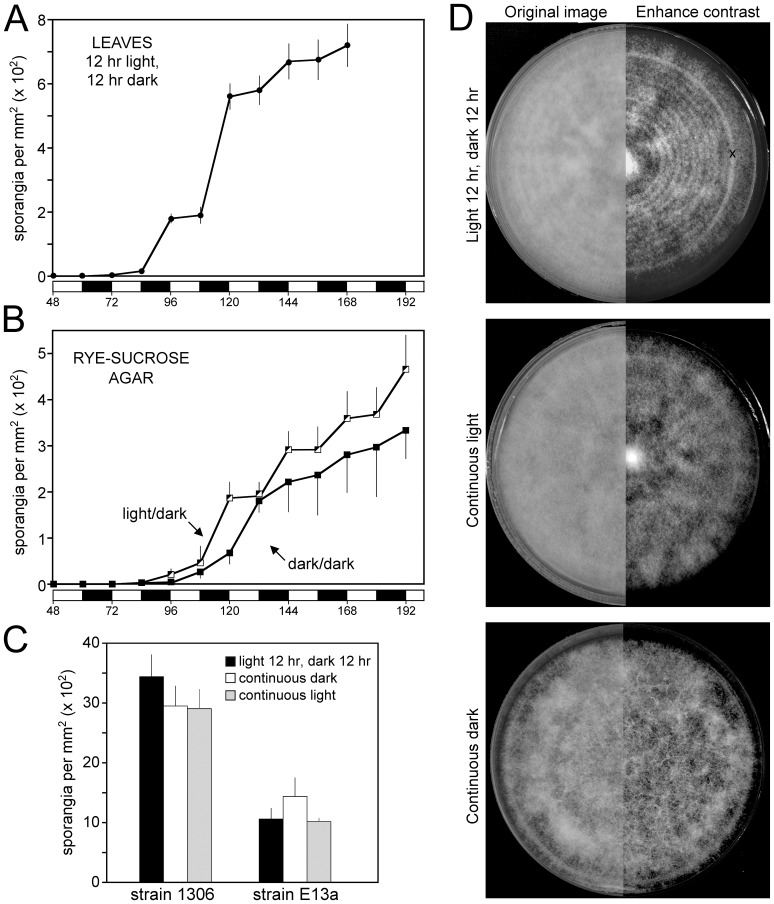
Effect of light on asexual sporulation in *P. infestans*. **A,** Sporulation of strain 1306 on tomato leaves incubated at 18°C with constant humidity and a 12 hr light/12 hr dark cycle, as indicated by the bars at the base of the panel. **B,** Sporulation of strain 1306 on rye-sucrose agar in plates exposed to a 12 hr light/12 hr dark cycle, or continuous darkness. **C,** Sporulation of strains 1306 and E13a on plates exposed to a light/dark cycle, continuous darkness, or continuous light, after 8 days. **D,** Strain 1306 grown under light/dark cycle, continuous darkness, or continuous light. The right side of each image has been contrast-enhanced to accentuate the growth rings in the light/dark regime. The “x” in the top panel is a ridge on the petri dish.

To confirm prior reports of the effect of light and obtain materials for further experimentation, tomato leaflets incubated under a 12 hr light/12 hr dark cycle were inoculated with strain 1306 of *P. infestans* under constant temperature (18°C) and humidity (98% relative humidity). Sporulation began near the end of the fourth day and occurred primarily during the dark periods of days four, five, and six; few sporangia formed in the light (Fig. 1A). The predominance of nocturnal sporulation was also observed on rye-sucrose agar, where sporangia first appeared after four days (Fig. 1B). Cultures in continuous darkness also began to sporulate after four days, but without the periodicity of samples exposed to the light/dark cycle. In studies of strain 1306 and a second strain, E13a, no consistent difference was observed in the total amount of sporangia formed by cultures exposed to light/dark, constant light, or constant darkness regimes for eight days (Fig. 1C). Light therefore appeared to suppress sporulation, but only temporarily.

Besides affecting the timing of sporulation, light influenced the morphology of cultures. As shown in Fig. 1D, cultures exposed to the light/dark cycle showed a banded pattern, with rings formed every 12 hr. The denser rings were formed in the dark phase, and represent zones with more aerial hyphae, upon which sporangia normally form. However, sporangia were present throughout the culture.

### Validation of genes marking sporulation-induced transcription

**Figure 2:**
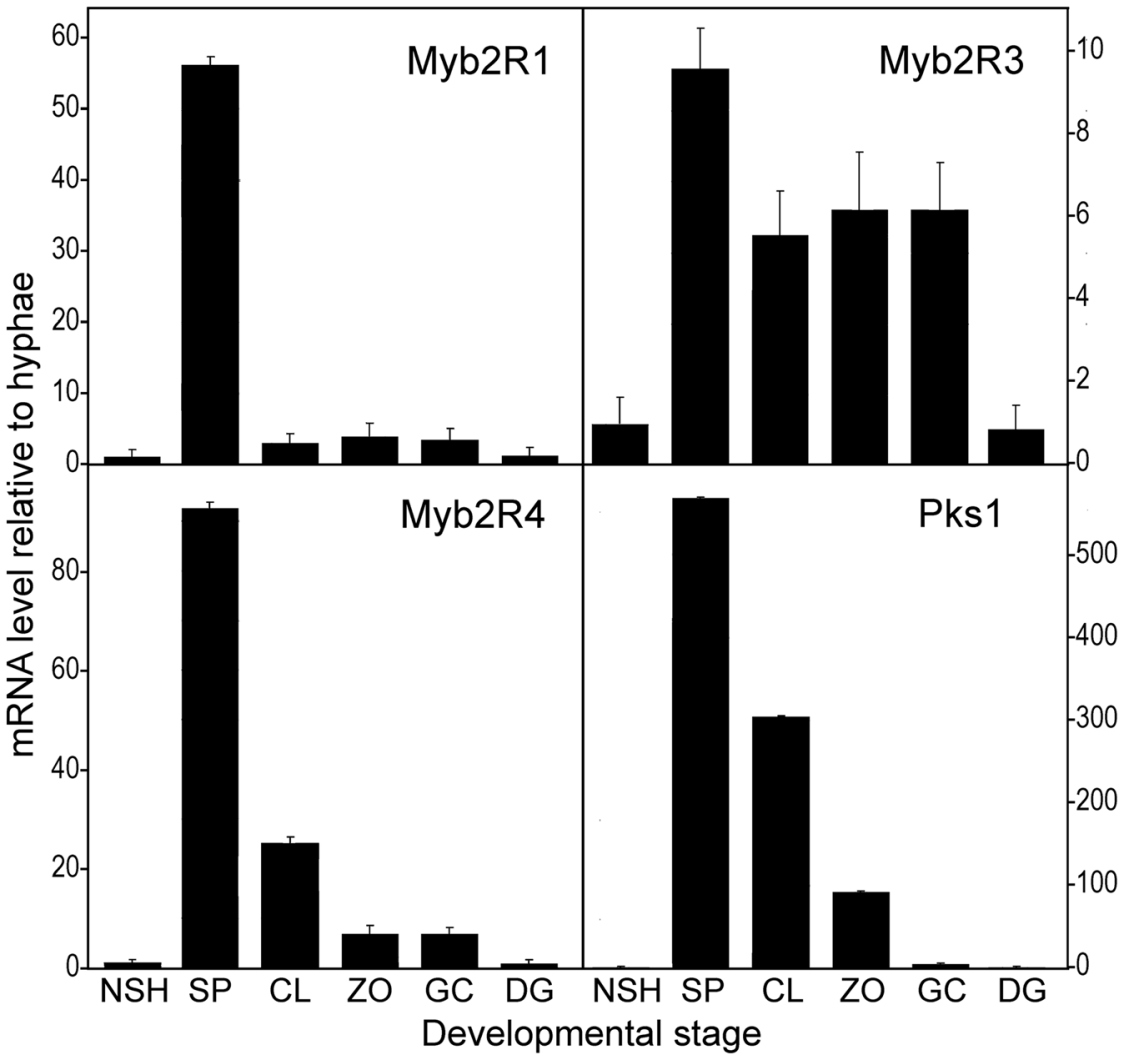
Expression patterns of markers for sporulation-associated transcription. Shown are three Myb genes *(Myb2R1, Myb2R3, Myb2R4)* and *Pks1* in six developmental stages, determined by RT-qPCR. The stages are nonsporulating hyphae (NSH), sporangia (SP), sporangia chilled to induce cleavage into zoospores (CL), motile zoospores (ZO), germinated zoospore cysts (GC), and directly germinating sporangia (DG). Error bars represent range of two biological replicates from RT-qPCR.

To help study the interplay of light, sporulation, and transcriptional regulation, four genes with potential roles in spore development were selected for analysis. These were reported previously to be sporulation-induced [22], [32]. RT-qPCR was used to confirm that conclusion, and measure expression in other life stages (Fig. 2). The gene encoding the Myb TF named in our prior study as Myb2R3 [22], which corresponds to PITG_06748 in the Broad Institute genome database, produced modest levels of mRNA in nonsporulating cultures, but high amounts in sporangia and subsequent stages such as zoospores and germinating zoospore cysts. Genes encoding Myb TFs Myb2R1 and Myb2R4 (PITG_01056, PITG_08755) and protein kinase Pks1 (PITG_10884; [33]) exhibited very little mRNA in nonsporulating hyphae, large amounts in sporangia, and declining levels after zoospore release.

### Genes induced during sporulation *in planta* show several patterns

**Figure 3:**
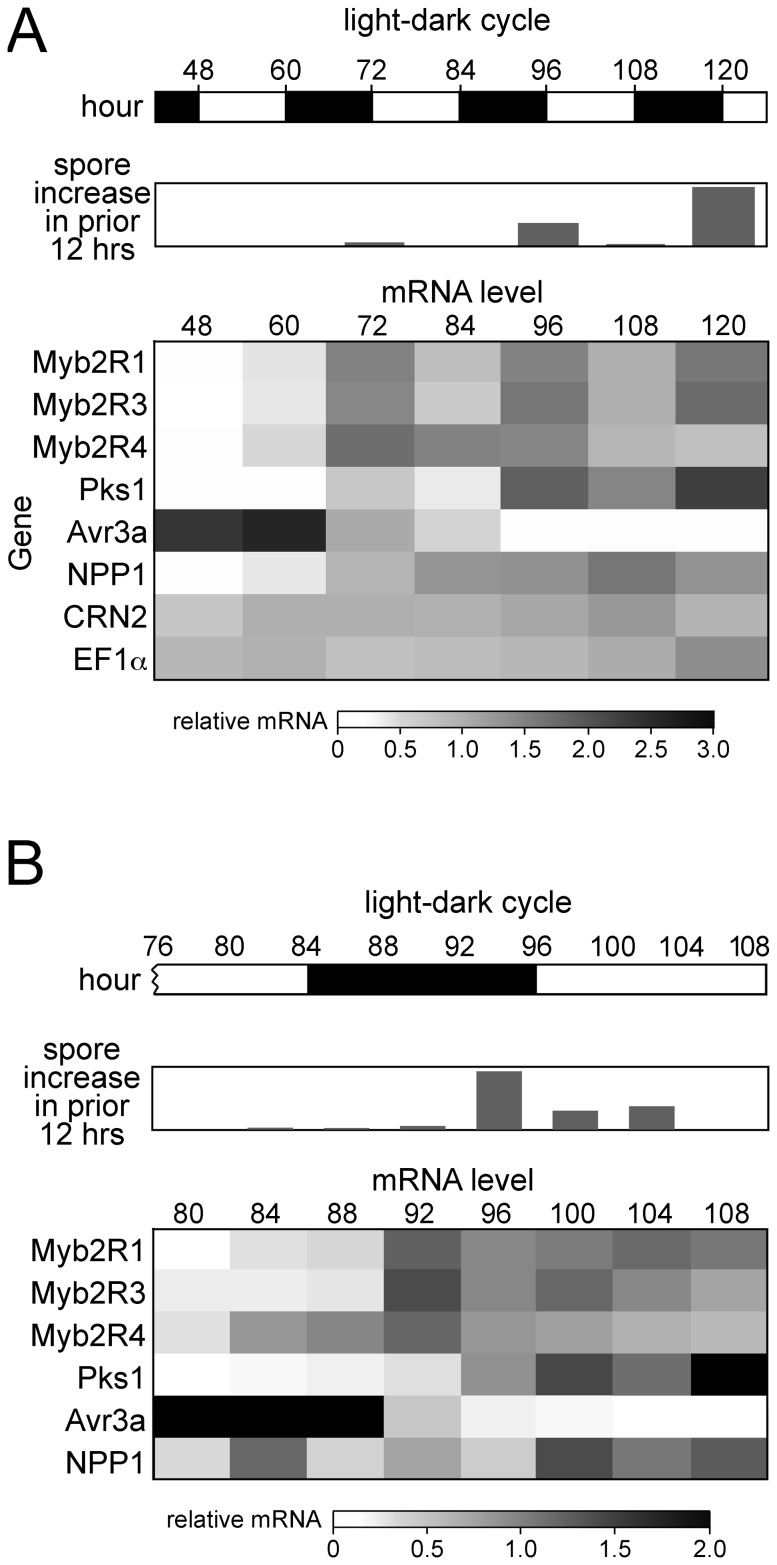
Sporulation and gene expression on leaves. Tomato leaflets were infected with strain 1306 and incubated at 18°C with constant humidity and a 12 hr light/12 hr dark cycle. **A,** increase in spore number over preceding 12 hr interval, and mRNA levels of *Myb2R1*, *Myb2R3*, *Myb2R4*, *Pks1* (sporulation-associated genes), *Avr3a* (linked to biotrophic growth), *NPP1* (linked to necrotrophic growth), and *CRN2* and *EF1α* (genes expressed at all stages). The heatmaps in this and later figures show per-gene normalized values, after normalization to the gene encoding ribosomal protein S3a. Spore numbers are relative values. **B,** Same as panel A, but with timepoints every 4 hr between 76 and 108 hr. Numerical RT-qPCR data are shown in Table S3.

Expression of the genes in *P. infestans*-infected tomato leaflets exposed to a 12 hr light/dark cycle were measured by RT-qPCR (Fig. 3A). Temperature and humidity were constant during this experiment, as in all work in this paper.

Levels of *Myb2R1*, *Myb2R3*, *Myb2R4*, and *Pks1* mRNA were low in leaflets prior to sporulation (48 hr timepoint) and increased just before sporangia appeared. However, the genes exhibited varying kinetics. *Myb2R1* and *Myb2R3* mRNA rose slightly during the 12 hr period of light preceding sporulation (60 hr), and more during the next dark interval, when sporangia first formed (72 hr). Their mRNA levels then oscillated, declining during each subsequent light period (84 and 108 hr) and rising during each dark period (96 and 120 hr). *Pks1* mRNA displayed a similar trend, but with a larger progressive increase during each period of sporulation than *Myb2R1* and *Myb2R3*. A distinct pattern was observed for *Myb2R4*. Its transcripts also began to rise at 60 hr, just prior to sporulation. However, *Myb2R4* showed no light/dark periodicity. *Myb2R4* mRNA peaked at 72 hours and fell progressively during the rest of the experiment, while mRNAs of the other genes generally increased along with the number of sporangia.

Several genes not associated with sporulation were measured as controls, as shown in Fig. 3A. *Avr3a* (PITG_14371) and *NPP1* (PITG_16866) have been described as markers for the biotrophic (early) and necrotrophic (late) stages of infection, respectively [34]. Such patterns were also observed here, but without the oscillations seen for the sporulation-associated genes. *CRN2* (PITG_17199, encoding an effector-like protein) and *Tef1* (PITG_09349, encoding elongation factor-1α) have been described as being expressed throughout the life cycle [25], [34], and here they also showed little variation. This confirms that the fluctuations in sporulation-associated mRNAs were not artifacts of the gene used to normalize RT-qPCR, which was PITG_11766 and encoded ribosomal protein S3a.

A study with additional timepoints provided more insight into when transcriptional changes and sporulation occurred *in planta* (Fig. 3B). Timepoints were analyzed every 4 hr between 80 and 108 hr post-infection, spanning most of two light periods and one dark period. Most sporulation occurred in the last 4 hr of the night phase, between 92 and 96 hr. *Myb2R1* and *Myb2R3* mRNA showed slight increases at 84 to 88 hr, and then jumped to much higher levels by 92 hr, just before most sporangia appeared. *Myb2R4* mRNA in contrast exhibited its major increase by 84 hr, 4 to 8 hr before the other genes. *Myb2R4* transcripts also declined faster during the next light period than those of *Myb2R1* and *Myb2R3*.

The experiments shown in Fig. 3 were performed on detached leaflets, in replicated experiments. Similar results were also observed in whole plants, where sporulation also occurred near the end of the dark period (not shown). For example, *Myb2R1* and *Myb2R3* mRNAs also oscillated with light and sporangia count in the whole-plant experiments. *Myb2R4* mRNA also reached its peak earlier than that of *Myb2R1* in the whole-plant studies, and showed little diurnal fluctuation.

### Influence of light on transcription and sporulation on artificial media

**Figure 4:**
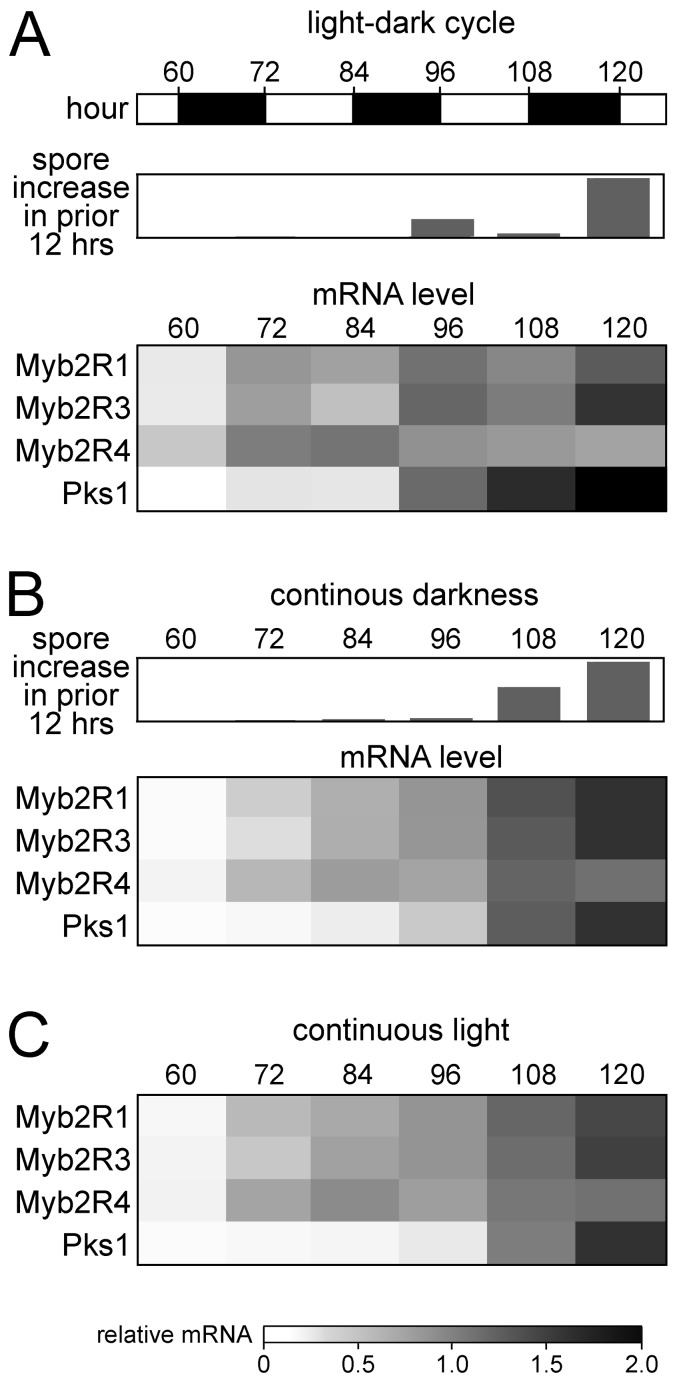
Sporulation and gene expression on rye-sucrose agar. **A,** Cultures of strain 1306 exposed to a 12/12 hr dark cycle, showing the relative increase in sporangia and mRNA levels of four sporulation-associated genes. **B,** Cultures exposed to continuous darkness. **C,** Cultures exposed to continuous light. Gene expression and sporulation data are presented as in Fig. 3. Numerical RT-qPCR data are shown in Table S3.

Trends similar to those detected *in planta* were seen in *P. infestans* cultured on rye-sucrose agar (Fig. 4a). *Myb2R1* and *Myb2R3* mRNA began to rise prior to the appearance of sporangia, had a local peak at 96 hr coincident with the first wave of sporulation, dipped slightly during the next light period, and then rose along with sporulation during the next dark period. As was the case *in planta*, the relative rise in *Myb2R4* mRNA by 72 hr on rye-sucrose media exceeded that of *Myb2R1* and *Myb2R3*. Moreover, *Myb2R4* mRNA started to fall by 120 hr, when *Myb2R1* and *Myb2R3* transcripts were rising. Also, *Myb2R4* mRNA did not exhibit light/dark oscillation, unlike *Myb2R1* and *Myb2R3*.

Contrasts between *Myb2R4* and the other sporulation-associated genes were also observed in cultures experiencing continuous darkness (Fig. 4B) or light (Fig. 4C). In both cases, the level of *Myb2R4* mRNA rose faster than the transcripts of *Myb2R1*, *Myb2R3*, and *Pks1*. Furthermore, *Myb2R4* mRNA started to decline by 120 hr, while mRNAs of the other genes were still rising. During the continuous dark or light regimes, there was no evidence for 12 hr periodicity in these mRNAs or sporulation.

### Myb2R4 binds the *Myb2R1* promoter

**Figure 5:**
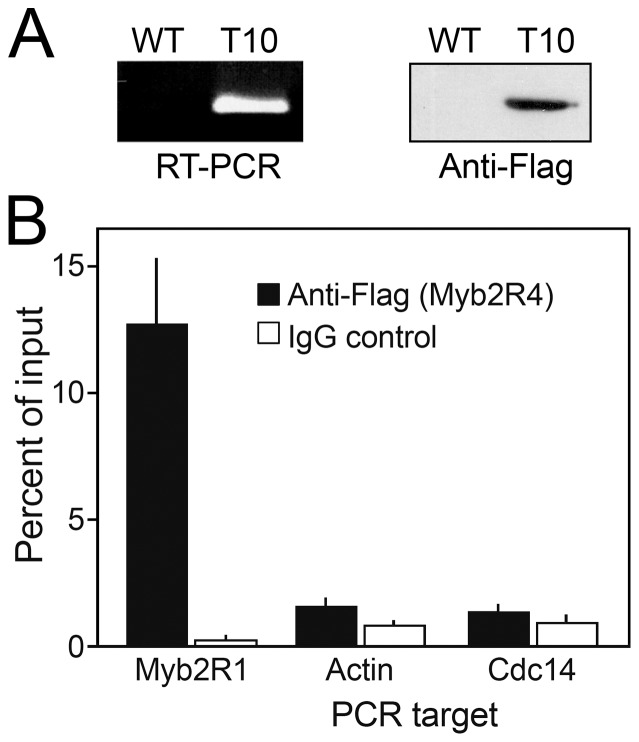
Chromatin immunoprecipitation (ChIP) indicates that Myb2R4 binds the *Myb2R1* promoter. **A,** Verification of expression of FLAG-tagged Myb2R4 in *P. infestans* transformant T10 by RT-PCR (left) and immunoblot analysis (right). **B,** ChIP analysis using the FLAG-tagged strain. Complexes from cross-linked chromatin were purified using anti-FLAG or control IgG, and subjected to qPCR using primers targeted to 250-nt promoter regions of the indicated genes.

The *Myb2R1* promoter bears two copies of a motif that matches the consensus binding site of Myb TFs [22]. Since *Myb2R4* is induced soon before other sporulation-induced genes such as *Myb2R1*, we considered whether Myb2R4 protein might bind the *Myb2R1* promoter *in vivo* to activate transcription. Chromatin immunoprecipitation (ChIP) studies confirmed that this was the case (Fig. 5). To accomplish this, we generated a *P. infestans* transformant expressing FLAG-tagged Myb2R4 driven by the constitutive *ham34* promoter (T10; Fig. 5A). Chromatin was precipitated using anti-FLAG antibody or control IgG, and the resulting DNA was subjected to qPCR using promoter-targeted primers. The *Myb2R1* promoter signal was 12 times stronger in the anti-FLAG sample compared to the IgG control (Fig. 5B). In contrast, little enrichment was observed in the anti-FLAG sample for promoters of other genes such as PITG_09284, which encodes an actin-like protein, and PITG_18578, which encodes a protein phosphatase.

### Gene silencing suggests that Myb2R3 regulates sporulation

Due to the potential role of *Myb2R4* in regulating genes early during spore formation, we tested the effect of silencing it and genes encoding other Myb TFs. Gene disruption has not yet proved feasible in *Phytophthora*, but DNA-directed RNAi using hairpin constructs in stable transformants can yield success [28]. This technique was thus applied to eight Myb genes that we described previously in *P. infestans*, including three expressed in nonsporulating hyphae and sporangia *(Myb2R5, Myb3R1, Myb3R3)* and five induced during sporulation or in spores *(Myb2R1, Myb2R3, Myb2R4, Myb3R5, Myb3R7)*; wild-type expression patterns of genes not shown in Fig. 2 are described in Xiang et al. [22].

**Figure 6:**
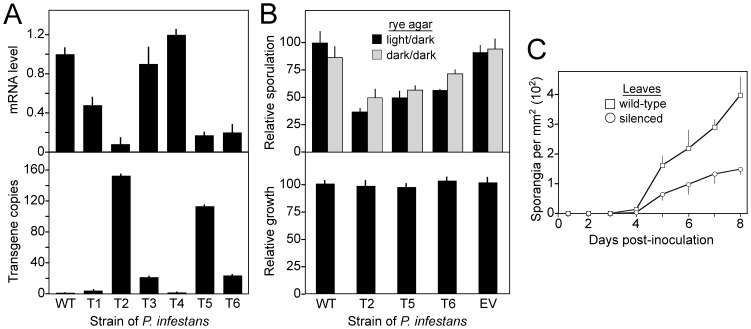
Silencing *Myb2R3*. **A,** Characteristics of six transformants (T1 to T6) and wild-type (WT). Top panel shows levels of *Myb2R3* mRNA in sporulating hyphae, indicating that T2, T5, and T6 show the strongest silencing. Lower panel denotes *Myb2R3* copy number, based on qPCR using primers detecting both the native gene and transgene. Similar results were obtained using primers for the *Ste20* intron that is between the sense and antisense regions of the hairpin construct (not shown). **B,** Sporulation and growth rates of transformants after 8 days in cultures exposed to a 12 hr light/dark cycle or continuous darkness. T2, T5, and T6 are the silenced transformants, and EV is a control transformant obtained using empty vector. **C,** Sporulation of wild-type and silenced transformants on tomato leaflets exposed to a 12 hr light/dark cycle.

We generated an average of 47 transformants for each gene, and used semiquantitative RT-PCR to identify potential knockdowns and RT-qPCR for confirmation. Convincing knock-downs were observed only for *Myb2R3*, as shown in Fig. 6A for six representative transformants. Three (T2, T5, and T6) have 5–20% levels of Myb2R3 mRNA compared to wild-type.

Since gene silencing in *Phytophthora* is not well-characterized, we also measured copy numbers of the *Myb2R3* transgene in those six transformants to help learn why only some hairpin-derived transformants exhibit silencing. There was a strong correlation (*R* = 0.68) between the level of silencing and copy number (Fig. 6A, lower panel). For example, transformants T2 and T5 had the highest copy numbers and greatest reductions in *Myb2R3* mRNA. The range in copy numbers is similar to that seen in a prior study in *P. infestans*, which detected 1 to 130 transgene copies per nucleus with a median of 20 [28].

The three strains silenced for *Myb2R3* were checked for abnormalities in growth and development. Each exhibited about 2-fold less sporulation than wild type on rye-sucrose media and *in planta* (Fig. 6B, C). This is consistent with a role of *Myb2R3* as a developmental regulator. Unaffected by silencing were radial growth rates on rye-sucrose media (Fig. 6B, lower), lesion expansion *in planta*, and spore germination (not shown).

### Ectopic expression of some Mybs impairs growth and is unstable

Since gene silencing did not succeed for most Myb genes, we also attempted over-expression. This was done for ten genes using plasmids designed to produce each Myb with a C-terminal FLAG tag, driven by the *ham34* promoter. Since *ham34* is strong and constitutive [35], it was expected that many transformants would express higher levels of these proteins than wild-type. Seven genes were successfully expressed in this manner. These included four sporulation-induced genes *(Myb3R2, Myb2R1, Myb2R3, Myb2R4)*, one expressed in most life stages *(Myb3R6)*, one transcribed primarily in hyphae and sporangia *(Myb2R5)*, and one expressed in germinating sporangia and zoospores *(Myb3R5)*.

**Figure 7:**
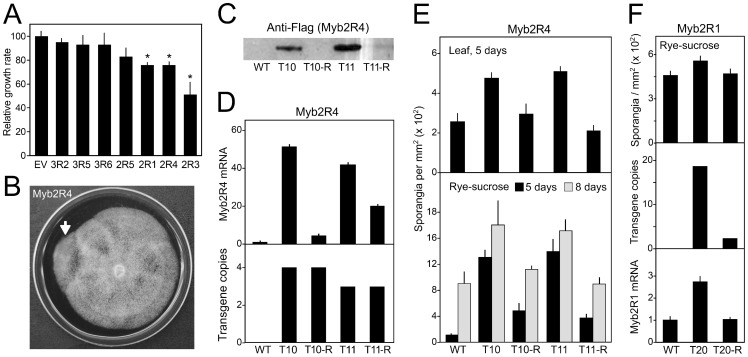
Over-expression of Myb genes. **A,** Growth of transformants expressing FLAG-Myb constructs. Values are normalized to empty vector (EV) controls; asterisks mark values significantly different from controls, and error bars reflect standard deviation between multiple transformants. **B,** Example of sectoring in a transformant over-expressing *Myb2R4*, with the arrow indicating a zone of more rapid growth. **C,** Loss of expression of *Myb2R4* transgene. Indicated are two transformants (T10, T11) and derivatives which lost or reduced expression (T10-R and T11-R, respectively). **D,** *Myb2R4* mRNA levels in hyphae (top panel, normalized to wild-type) and transgene copy number (lower panel) in the strains from panel C. **E,** Sporulation rate on leaves and rye-sucrose agar in the strains from panel C. **F,** Transformant over-expressing *Myb2R1* (T20) and a derivative (T20-R) with reduced transgene expression level and copy number.

Strains over-expressing each of the seven genes produced sporangia capable of releasing zoospores and infecting plants (not shown). However, compared to wild-type and empty vector controls, strains over-expressing *Myb2R1*, *Myb2R3*, and *Myb2R4* showed reduced radial growth on rye-sucrose media (Fig. 7A). Further and more quantitative phenotypic analyses were complicated by the instability of transgene expression. Appearing in cultures were faster-growing sectors (*e.g.* Fig. 7B), in which transgene expression was reduced. Examples involving *Myb2R4* are shown in Fig. 7C; T10 and T11 are transformants that make FLAG-tagged Myb2R4, while T10-R and T11-R are derivatives in which transgene expression was reduced and the slow-growth trait lost. Although T10-R and T11-R displayed diminished expression of the transgene, its copy number was unchanged (Fig. 7D).

We also molecularly characterized a transformant exhibiting instability of expression of FLAG-tagged Myb2R1. This is illustrated in Fig. 7F, which shows transformant T20 and a non-expressing revertant, T20-R. Interestingly, transgene copy number was reduced from 18 copies in T20 to two in T20-R. The slow-growth trait also disappeared in the revertant.

Among single-zoospore (single nuclear) derivatives of the unstable transformants, some maintained expression of both the Myb transgene and the *nptII* marker, which had been used to select the original transformants. Others expressed only *nptII*, even though both genes had been introduced on the same plasmid. Single-nuclear derivatives that expressed both transgenes sometimes became unstable in later generations. Therefore, the phenomenon can not be generally explained by the initial transformants being heterokaryons of transformed and nontransformed nuclei. Instead, there appears to be selective pressure to inactivate some Myb transgenes, since over-expression can be deleterious. A similar principle may explain why we failed to obtain strains over-expressing *Myb2R2*, *Myb3R4*, or *Myb3R7*, even after screening 75 transformants.

### Ectopic expression suggests that *Myb2R4* regulates sporulation

Transformants over-expressing *Myb2R4* from the *ham34* promoter (T10 and T11) showed about two-fold higher rates of sporulation than wild-type controls (Fig. 7E). The connection between ectopic expression and increased sporulation is strengthened by the fact that strains T10-R and T11-R, which lost most transgene expression, reverted to normal sporulation.

A related phenotype was observed in a transformant over-expressing *Myb2R1* (Fig. 7F). The over-expressing strain (T20) showed a 20% increase in sporulation relative to wild-type, while a revertant (T20-R) returned to normal. Although conclusions related to *Myb2R1* are tempered by the fact that we could identify only a single over-expressing strain, we present that data to contrast two mechanisms of transgene instability: apparent epigenetic suppression in T10-R and T11-R, and transgene excision in T20-R.

### Over-expressing *Myb2R4* upregulates *Myb2R1*

**Figure 8:**
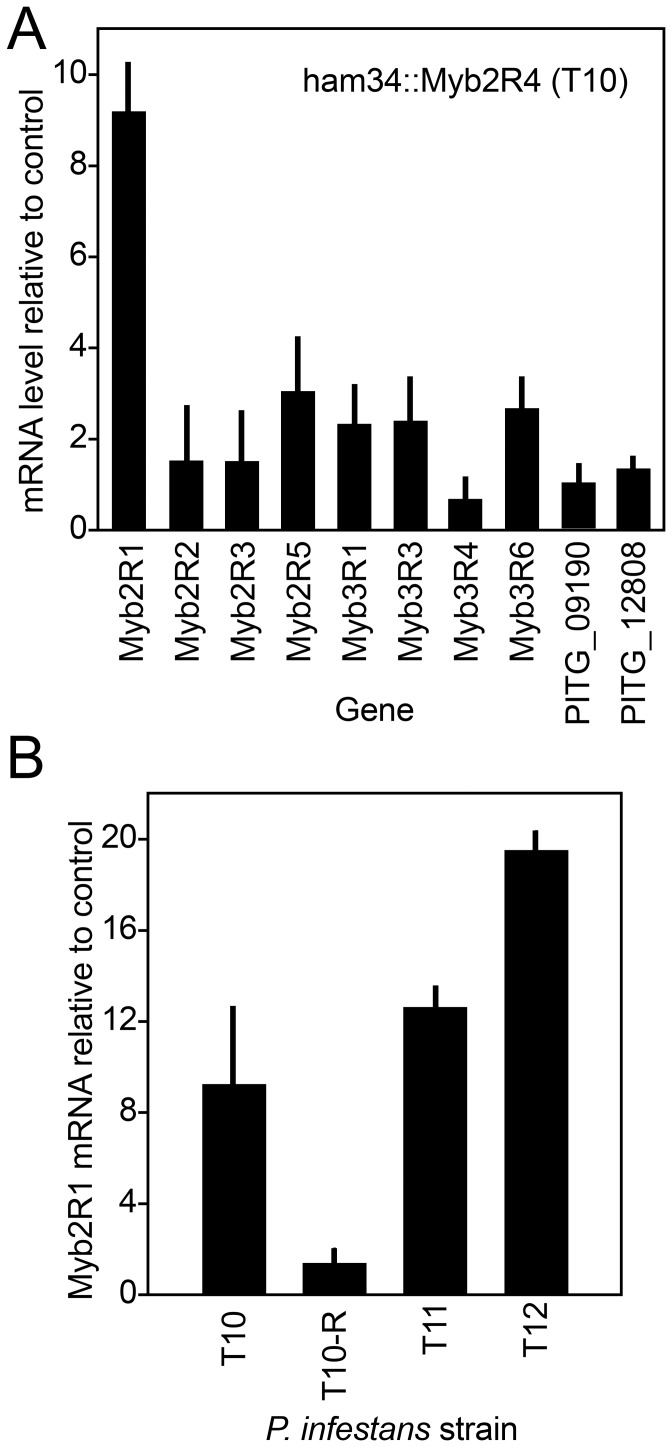
Over-expression of *Myb2R4* upregulates *Myb2R1*. **A,** RT-qPCR of Myb2R4::FLAG transformant T10 using primers against the indicated genes against mRNA from sporulating hyphae. **B,** *Myb2R1* mRNA levels in three *Myb2R4*-over-expressing transformants (T10, T11, T12) and the derivative of T10 with reduced transgene expression (T10-R).

To test the idea implied by our ChIP results that Myb2R4 controls the transcription of *Myb2R1*, we assessed if over-expressing Myb2R4 increased mRNA levels of the latter. In transformant T10, *Myb2R1* mRNA was indeed higher than an empty vector strain by about 9-fold (Fig. 8A). Slight increases were observed for several other Myb genes *(Myb2R2, Myb2R3, Myb2R5, Myb3R1, Myb3R3, Myb3R6)*, but this is probably because these genes are sporulation-induced and T10 produces double the normal amount of sporangia. As controls, we observed normal mRNA levels for genes that are not sporulation-induced. These were PITG_09190, which encodes a bZIP TF, and PITG_12808, which encodes an amino acid transporter.

We confirmed the connection between *Myb2R4* over-expression and *Myb2R1* stimulation by examining two additional transformants that over-express *Myb2R4*, T11 and T12, and the T10-R revertant (Fig. 8B). *Myb2R1* mRNA levels ranged from 8 to 20-fold higher than normal in the three over-expressing transformants (T10, T11, T12). Furthermore, *Myb2R1* transcripts returned to wild-type levels in T10-R.

### Over-expressing *Myb3R6* weakens sporangia dormancy

**Figure 9:**
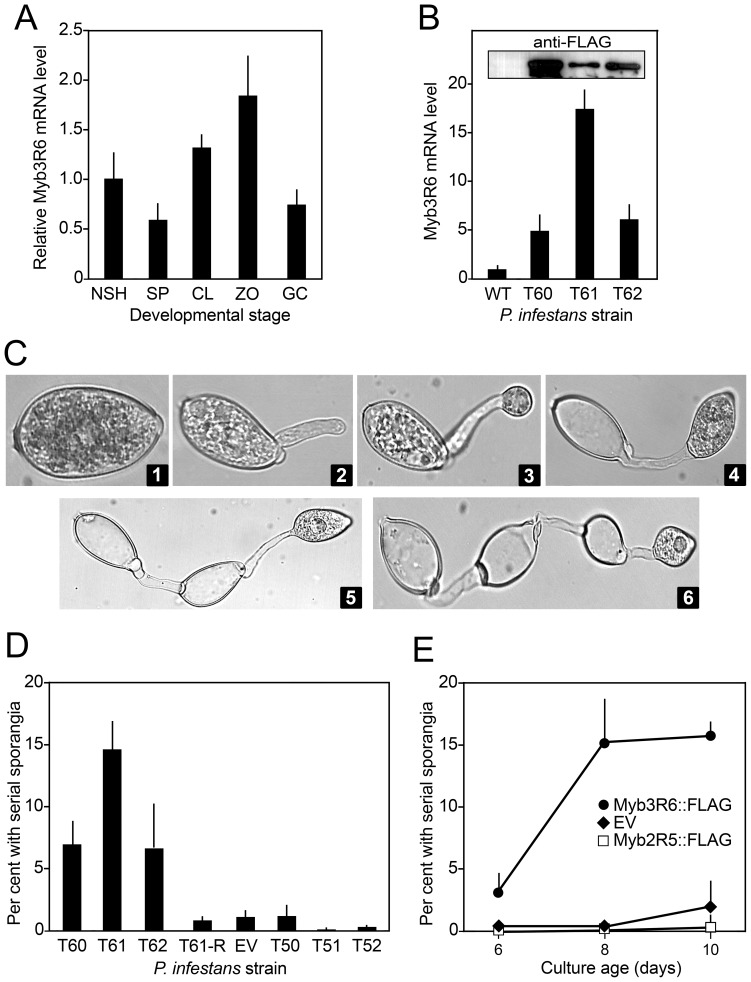
Serial sporulation induced by over-expression of Myb3R6. **A,** mRNA levels of *Myb3R6* in wild-type, using the same developmental stages as in Fig. 2. Error bars represent standard deviation from three replicates. **B,** mRNA levels in three over-expressing transformants (T60, T61, T62) and wild-type (WT). **C,** inferred stages of aberrant germination observed in sporangia harvesting from rye-sucrose agar cultures. Images show sporangium of normal appearance (panel 1), sporangium extending germ tube (panel 2), secondary sporulation (panels 3,4), and formation of a third (panel 5) and fourth sporangium (panel 6). **D,** Percent of sporangia exhibiting serial sporulation from 8-day cultures of strains expressing Myb3R6::FLAG (T60, T61, T62), a non-expressing revertant of T61 (T61-R), an empty vector transformant (EV), and strains expressing Myb2R5::FLAG (T50, T51, T52). **E,** Age-dependence of serial sporulation. Data are from a Myb3R6::FLAG transformant, a Myb2R5::FLAG transformant, and an empty vector transformant.

Unlike the Myb genes mentioned earlier, *Myb3R6* is transcribed in all developmental stages (Fig. 9A). We analyzed three transformants over-expressing the Myb3R6::FLAG fusion from the *ham34* promoter, that made from 5 to 15 times the normal level of *Myb3R6* mRNA (Fig. 9B). Each showed an increased propensity towards premature direct germination followed by repeated (serial) sporulation, as illustrated in Fig. 9C. When sporangia are first harvested from a culture plate they normally appear as in Fig. 9C, panel 1: metabolically active and undesiccated, but ungerminated. This was not the case for many sporangia from the over-expressing transformants. As shown in Fig. 9C, panels 2 to 6, many sporangia in 8-day cultures had undergone precocious germination and serial (secondary, tertiary, and quaternary) sporulation, as evidenced by the presence of multiple interconnected germ tubes and sporangia. Although involving a different germination pathway, this is similar in concept to the ability of some species of *Phytophthora* to resporulate after zoospore release [36].

Serial sporulation was observed in 7 to 15% of sporangia harvested from hyphae of transformants over-expressing *Myb3R6* (T60, T61, T62; Fig. 9D). The phenomenon does occur in wild-type *P. infestans*, but only affects <1% of sporangia under standard growth conditions. The same low frequency of abnormal sporangia was seen in transformants made with the empty expression vector or expressing other transgenes such as *Myb2R5* (T50, T51, T52; Fig. 9D). The propensity for serial sporulation increased with culture age (Fig. 9E), but was always much higher in the transformants over-expressing *Myb3R6*.

## Discussion

Like many microbes, *P. infestans* uses environmental cues to optimize asexual spore production. This study has provided more details about the influence of light on the sporulation; identified Myb2R4, Myb2R1, and possibly Myb2R3 as regulators; and used ChIP and over-expression experiments to demonstrate a direct link between Myb2R4 and Myb2R1. Interestingly, all *P. infestans* genes encoding Myb2 (R2R3 domain) proteins are sporulation-induced, as are some encoding Myb3 (R1R2R3) proteins [22]. We do not claim that Myb proteins exclusively orchestrate transcription during the transition from hyphae to sporangia in *P. infestans*, but there are precedents across multiple eukaryotic kingdoms for developmental events being regulated by the concerted action of members of the same TF family [37]–[39]. Multiple regulators must be involved since sporulation involves many sub-stages such as sporangiophore emergence, coordinated nuclear divisions, development of sporangial initials, papilla formation, establishment of dormancy, etc. [15], [40].

Other TF families plus post-transcriptional regulators likely also regulate sporulation. Many protein kinases and some phosphatases are known to be induced at this stage, for example [25], [41]. Indeed, a recent study in the related species *Phytophthora sojae* demonstrated that a protein kinase influenced the expression of *PsMyb1*, which is an ortholog of *Myb2R3* of *P. infestans* [42]. While our analysis found that each of three *P. infestans* transformants silenced for *Myb2R3* were reduced in sporulation, the other study examined one *P. sojae* strain silenced for *PsMyb1* and found that it made more sporangia than wild-type. It is unclear if all *PsMyb1*-silenced strains would also have that trait, but the *P. infestans* and *P. sojae* orthologs may operate dissimilarly as the two species sporulate differently. For example, starvation is needed to induce sporulation in axenic cultures of *P. sojae*, but not in *P. infestans*. Also, the sporangia of *P. infestans* are deciduous, *i.e.* freely separable from sporangiophores, unlike those of *P. sojae* [13]. There are also differences in gene expression patterns: while *Myb2R3* is sporulation-induced, levels of *PsMyb1* mRNA are not much higher in sporulated than nonsporulating cultures [42]. Moreover, while *Myb2R3* transcripts remained abundant in *P. infestans* zoospores, *PsMyb1* mRNA was reported to be very low in *P. sojae* zoospores.

Prior researchers noted that *P. infestans* produces most sporangia at night, which was attributed to its high relative humidity [14], [15]. Nocturnal sporulation presumably benefits *P. infestans* since its sporangia lack pigments for blocking ultraviolet light and may be more prone to mid-day desiccation [43]. After dawn, fluctuations in temperature and humidity occur that help detach sporangia from sporangiophores, and water films conducive to zoosporogenesis are likely to be present [44], [45]. Light maximizes night sporulation by inhibiting daytime development, but humidity seems more critical; once a culture achieves “sporulation competence”, development proceeds only if humidity is above 85–90% [14], [46]. Unlike low humidity, light only temporarily delays sporulation, which will occur in cultures receiving continuous illumination. It should be noted that our studies do not address whether light entrains a circadian clock.

Past reports of the effect of light in *Phytophthora* are conflicting, with light said to inhibit, stimulate, or have no influence on asexual sporulation (rev. in [17]). While some disparity in the literature might be explained by procedural issues, it is also possible that different species may have evolved to respond in varying ways. As noted above for *P. infestans* and *P. sojae*, not all members of the genus sporulate in same manner. *P. infestans* is largely a foliar pathogen and thus may benefit from nocturnal sporulation, but sporulation in the light may promote the aerial dissemination of root-infecting species. Light is known to regulate the balance between asexual and sexual spore formation in some homothallic *Phytophthora* [47]. In *P. infestans*, which is heterothallic, we are aware of three controlled studies of the effect of light on asexual sporulation, although none used day/night cycles as employed in our study. These reported that 10 min of light stimulated sporulation [48], continuous daylight reduced sporulation [49], and inhibition resulted from 24 hr of continuous blue light, but not green or red [50].

That blue is the bioactive wavelength may help indicate what *P. infestans* molecule is the receptor. Of the six types of known photoreceptive proteins [12], only the blue light receptors known as cryptochomes are predicted in *P. infestans*. Cryptochromes belong to the photolyase/cryptochrome family of flavoproteins and are found in archaea, eubacteria, and eukaryotes [51]. The family is classified phylogenetically and biochemically into cyclobutane pyrimidine dimer (CPD) photolyase, plant CRY, animal CRY, and CRY-DASH (*Drosophila*, *Arabidopsis*, *Synechocystis*, Human) groups [52]. The three putative *P. infestans* cryptochromes are encoded by genes PITG_01718, PITG_16100, and PITG_16104, and cluster in phylogenetic analyses with animal cryptochromes (Figs. S1, S2).

Light served a useful technical role in this study by facilitating the detection of differences in expression between sporulation-associated genes. Multiple stages of sporulation can be discerned in *P. infestans* by microscopy, including the emergence of sporangiophore initials, nuclear migration, sporangiophore elongation, nuclear division, swelling of sporangial initials, cytokinesis, and formation of the terminal papilla and basal septum [15], [40]. However, these are asynchronous under traditional unilluminated culture conditions, which makes dissecting the stages by RNA analysis impractical. In our light-regulated timecourses, development was spread over longer periods and more synchronous, which helped make it apparent that *Myb2R4* was induced earlier during sporulation than *Myb2R1*, *Myb2R3*, or *Pks1*.

The role of Myb2R4 as a regulator of sporulation was supported by our observation that *in vivo* it bound the promoter of *Myb2R1*. Myb2R4 over-expression also stimulated sporulation along with *Myb2R1* transcription. Demonstrating Myb2R4 function by stable gene silencing proved elusive, however. We also attempted a transient gene silencing method reported by another group, but without success [53]. Even the use of over-expression to discern Myb2R4 function was challenged by epigenetic events that impaired transgene expression. Loss of expression has also been reported for other transgenes in *P. infestans* [54], but appeared more problematic for Myb genes, perhaps since over-expression often negatively impacted fitness. With *Myb2R4* and *Myb2R1*, we nevertheless used the phenomenon to our advantage by correlating loss of transgene expression with reversion of increased sporulation phenotypes. It could be argued that over-expression indirectly stimulated sporulation, as a side-effect of reduced vegetative growth.

We observed that inverted repeat transformants with higher copy number were more prone to trigger gene silencing. Similar findings are reported in mammals and plants [55], [56]. High copy numbers are common in *P. infestans* transformants, as most contain tandem repeats of the transforming plasmids [57]. This may also help explain the reduction of transgene copies in some over-expressing strains, since recombination within the repeats could cause excision [58], [59]. This is distinct from losses of transforming DNA reported in the related species *Phytophthora parasitica*, where transgenes persisting as extrachromosomal units were not passed to all daughter nuclei [60]. Nevertheless, vigilance is required when testing transgenes in *Phytophthora*, particularly if a deleterious phenotype is conferred.

## Supporting Information

Figure S1 **Phylogenetic tree showing relationship between** ***P. infestans*** **cryptochromes and plant CRY, animal CRY, CRY-DASH, and CPD type 1 photolyase groups.** The latter four groups are defined as in Daiyasu et al. [52], including the presence of some fungal sequences in the animal CRY group. Also shown are one of the three *Phytophthora sojae* orthologs and one of two orthologs from *Albugo laibachii*, which are also oomycetes. Alignments were performed using MUSCLE and a PhyML tree developed using the SEAVIEW program.
(TIF)

Figure S2 **Structures of predicted cyptochromes from** ***P. infestans*** **.**
(TIF)

Table S1 **Corrected gene models.**
(PDF)

Table S2 **Primers used for PCR.**
(PDF)

Table S3 **Numerical values of RT-qPCR data shown in** **Figs. 3** **and** **4** **.**
(PDF)
