# Supplementary figures and images for: Myb Transcription Factors and Light Regulate Sporulation in the Oomycete Phytophthora infestans
Source: PLoS One. 2014 Apr 4;9(4):e92086. doi: 10.1371/journal.pone.0092086 (PMC3976263; doi:10.1371/journal.pone.0092086)

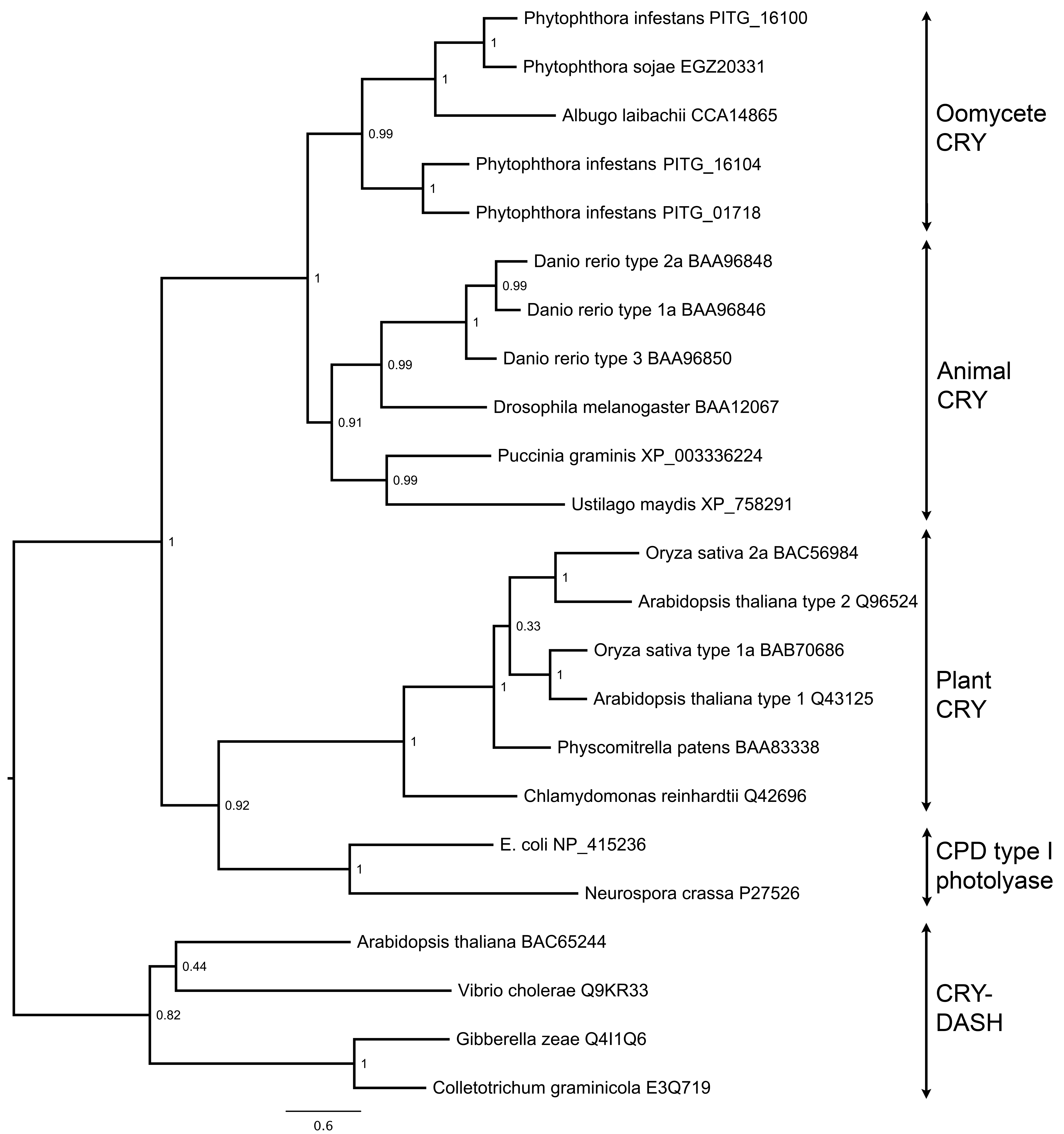

Supplement: Figure S1 — Phylogenetic tree showing relationship between P. infestans cryptochromes and plant CRY, animal CRY, CRY-DASH, and CPD type 1 photolyase groups. The latter four groups are defined as in Daiyasu et al. [52], including the presence of some fungal sequences in the animal CRY group. Also shown are one of the three Phytophthora sojae orthologs and one of two orthologs from Albugo laibachii, which are also oomycetes. Alignments were performed using MUSCLE and a PhyML tree developed using the SEAVIEW program. (TIF) [file pone.0092086.s001.tif]

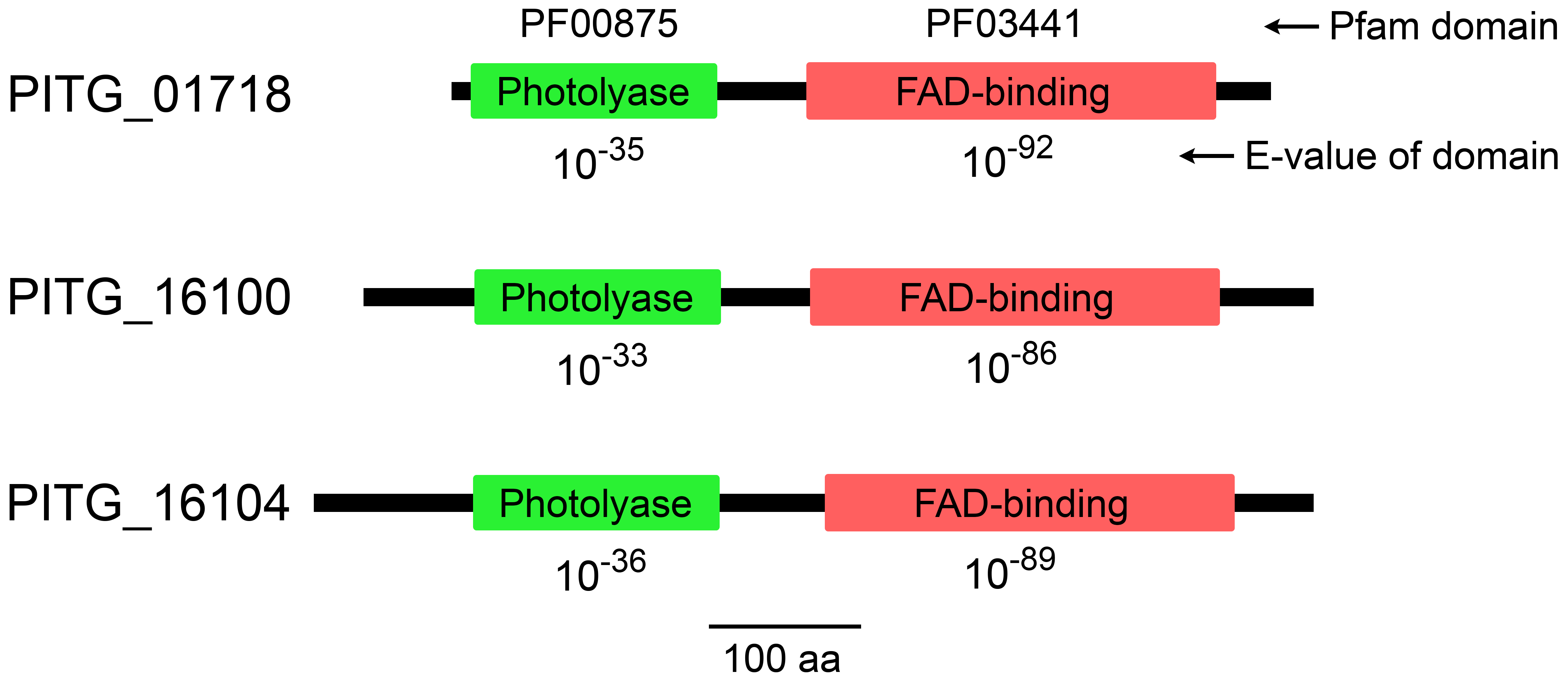

Supplement: Figure S2 — Structures of predicted cyptochromes from P. infestans . (TIF) [file pone.0092086.s002.tif]
