# Supplementary material for: Myb Transcription Factors and Light Regulate Sporulation in the Oomycete Phytophthora infestans
Source: PLoS One. 2014 Apr 4;9(4):e92086. doi: 10.1371/journal.pone.0092086 (PMC3976263; doi:10.1371/journal.pone.0092086)
Supplement: Table S1 — Corrected gene models. (PDF) [file pone.0092086.s003.pdf]

Table S1. Corrected gene models (coding sequences)

```
>PITG_1056
ATGGGCAAGCCGAGCAAGATGGACGGCCGTGTCAACGGCACGGGCTCCACGTACGAGCGCCGCGCCTGGACGCGCAAGGA
AGACGATGCTATCATCCGACTCGTAGAGGAGTACGGCACTAAGCGCTGGTCGGTCATATCGGACCACTTAAATGGGGAGA
ATCACGGTACGGAGCGGACAGGCAAGCAGTGTCGCACGCGATGGCTCAACCACCTGGACCCCACCATTAAGAAGGACCCT
TGGACTGCCGAGGAGGAACAGATCATCGAGGACGCACAGACGCGTCTCGGCAACAAGTGGGCGGAGATCTCCAAGCTGCT
GCCTGGCAGGACGGATAATGCCATCAAGAACCACTGGTACTCGTCCATGAGACGGACGATGCGTCGCATGGCGAAGCAGC
AGAACAAAACACTGGGCCAAGTGGCACGAAGCGGAGTAGGAAGCAACAAGGCCGCCAAGTTTGGTGTGAATGCGTCGCCC
GGCTCAATGGTGGAGCTGGCGCGAGACGCCATGAACGCTGGTCGTCAGGGTATGTCGTCCACACAGGCCTTAGTGTATAA
GGACTGCTACAACGCCCTGATCAAACAGGCAGAAGACGGAAAGGGACGTCAAGGGCAACGAAATGTTAATAAGAAACTCA
ACTCGAAGCGGAAACGCAAAGATCTGCGCATCTGCACGGGCAGTGAAAACAGCGGCATGTTCTTGCCAGATACGCCTCGA
CGTGTTCTACACACGCAGCTATTGCTGCAGCTCTTGAACAACGGAGAGGAGGATTTCGGTACATTCATCACGGTCGCCAG
CACAACCAACAAGAACAAGAAGCGGACTGCTCAGGGCAAGAGGAACGGAGTCAATGGAGCTGGCGTCGGTGAGTTTGGCG
GTGACCATGCATTTACCGAGATGGACGGATCCTTCCACGCCTTCGAGCACCTTGACATTGACTTTAACGAGGTACGCAGT
CGGTCC
>PITG_06748
ATGAACCGTAAGCTGGAGCTCATGGAGCTGGACAGCGGCGGCGCCAGCCCTAACAGCAGCAACAGTGGAAGCCCCAGTCC
GACTCTCGACCTGCTGCATCGCTCTGATTCGAGTAGCAGTAACCAGGGGGGTGAGATGCCTCTTGTCGGGGTTGCCCACA
ACCCAGACCTGAGTTATTTGAGGGCAACTGCAACACATGGAGGCAGCAGCACGACCAGCAGCAAGTATGGCAGCCCCGAG
GAGTCATTTGGTGAAGAGGACGAGGACAACAACAAGAAGACGGCGCGTCGCAAGACCGACTCCAACAGCAAGCGTCCGTG
GACACGCGAGGAGAATGACAAGCTCATGCAACTCGTCAAGCAGTACGGCGCCAAGCGCTGGTCTCTCATCGCCATGCACC
TGCCAGGCCGTGTCGGTAAGCAATGCCGTGAGAGATGGCACAACCACTTGAATCCGTCGGTCCGCAAGGACGCATGGACG
GCTGAAGAGGACTATGTCATCTTCGAGTGCCACAAGAATGTGGGCAACCAATGGGCCGAGATTTCCAAGATGTTGCCTGG
CAGGACGGACAATGCCATCAAAAACCGCTACTACTCGACCATGCGTCGCATGCAGAGGCAGTCAATCCGCAAAAAAGGTC
CCATGCGTGATGGCAAGAGCATCCGCGTGGCGTCAGTCACGTCGTCTCCCGTGCAAAACAACAACCAAATGGGTCCTGCT
CCCAGCCAGCGATCTTTGACTGGCATGCAACACCAGTTGCCACCGCAACAACAGCTTCCGCACCGAGGGGTGAGTTTCCA
ATCCACATATCAACGGCTCTTCTCGGAGGCCTCTGGGGACGCCGCACGCGATGGCAACTCGATAGTGGACTTTGAACGTT
CAAACATGATGTCCGCATCACTACGACAACCCACGATGGTCTACCCGCTAAACGGAGGTAGCTTTTCAAACGGGATGAAC
CCGGACTATAGTCGTCCCATGTCCATGACGTCGTCTCCCTGCTCGGTGTCCCCCGCCGATGATCTGAATCATAACAGCCA
GAACGAGACCTTTGACTACGTTCCGATGCAATCTTCGATCCAGCGTGTACGGTCATCTAGTCCGGTTGTGATGGGCACTC
CTCCTTCGACGTCAATAGGCATGATGAACTCACCTTACGGCTCGCCTGCAAGTCACATCCAACAGCAACAGCCTGGCAAC
TACATGATGGCAGGAAACCCATATTCTAACAACAACGTGCGTGGGATGTACTTTGGGACGTCTGGGCTTGCTCAGCAATA
CCGTCCTGATGCCCCTGTAAACGTCCCACACAAGCGCCTCCTCGACGTCCAGGGTTCACAACGTGATATGTGGAAGAATG
ATAGCCCTGTATCGGTAGCAGCACCGATATTCCGCGGTATGCCGGCGACGCACATGCAGCAAGGTCAAGTCAGCGAACCG
ATGGCATTGCAGCAGTCCAAGCCAGCTTCCATGCCACTGTATAGGCAAGCGCCGAATATGGGTCACTTCAACAGCATGGA
GCAGGTATGGACGGATGATGCATATCTG
>PITG_00988
ATGCAAAGCCTGCAGCATCTAAGCAGCTACAACAGCAACAGCGAGTTCGAGCCGCCGCAACTGGACCCTCCGACGCGTAA
AGAGCGCAAGCCTGTGTGCAAGTGGACGGAGAAGGAAGATCTCTTGATGCTCAAGCTCGTGCAGAAGTACGGCACGCGTC
ACTGGACTATCATCGGCACTAAACTGCCTGGCCGTAATGGCAAGCAATGCCGCGAAAGGTGGCACAACCAGCTTGACCCC
GCAATTCGCAAAGAGCCTTGGACACCCGAGGAGGAGCGCATCCTAAAGGAATTGCACGACAAGTTCGGCAACAAGTGGGC
GGAGATCGCCAAGATGTTGCCGGGCAGGACAGACAACGCCATAAAGAACCACTGGAACTCAAGCAAACGTCGTCTCAAGC
GCGGCTTCACTCCGACGTCCACCTCGCAACGCAAACGCCGTGATAGCTCCGGCTCGGACAGCTCTTCCAGCAGGGAAATT
TCAGAGATACCGTCGCCTGTGTTAGGTGGAGTCAACGACGTCTATCCACTCAGCACACTGCTCACGGACCAAGTCGACTT
TGCAAGTCTGTACCCGAACAATTACCTTCAATCTCCTCTTGCCATTCAAACAGCGCAACTCGGTAGTCCACAGATCTCTC
TGTGTTGGACCCCAACGGACGCGACGTACAACCGACTGAACGCCATGTGGAACATCCCCGCGATGCCTAATTGGGTTTCT
TCTGGCTTCAGCAACCCTCTGAACAAAGCTACGACGATTGAGCCGAGTAGAACTAAACTGGCGTCTGACCGAGCCCCCGC
CATGAACGGCAAACGTATTTTGGACGACACGATTGATGTTAGCAAGATACAGTCGCCGCTTACGAAGAAAGTGAAGAAAG
ACGACCCATCCCTGGAGATCCTGGCCAACGCGGCACTACTGCAGTCGATAGGCCACACGGTGTAG
>PITG_08755
ATGCAGCAGCCCGAGTGCGAGCCCCTCGCCTACCACGACGATATCACTCCTCCCAAGCCTGAAGACGTCAAGCAAAACAA
TGATGAGAGCGAAGTGCAGCAGCTGAGAGATCGTTTGGCAGCACAGGAGCTGTTGACACAGCAGCTACTAAAGCGCGAAC
AGGAGCGAGAGACCGAACGCGAGTTTATGAAGAGGAGCGTGCTGTCCTTACAGCAGGACCTTCTTCGTCTCATGAACATC
GTGGACCTGCAGATGCAGATCCCGTCCATGCACATGCAGATGCAGCTTTCGCCTATGGCTACAGGCGTCGAAATGGCGTT
TCGAGGACTCTCGCCGCGATTCCCAGCCAGAGGCTGCATCGGTCCAAGTCCAACGGGACACAGAGTAGCCAAGAAGCGAA
ATGCTGAGAGTGACGAGGCTCAAGCAGTGGCGGCATTGGTCTCGCCTGCACGCGATGCTGCGATGTTGATGGACATGTCA
CCGCAGAGTAAGCGCGTCAAGACGTCGTCCACGTCCAATAACAAGCTGAGTAAACGTCAGTGGAGCCCTGAGGAAGACGA
AGCATTGGAGCTCGCCATTCAATCAACGGGGGCCAACGATTGGTCGGCTATATCTCGACTGCTGCCTGGGCGTTGTGGGA
AACAATGTCGGGAGCGATGGGTGAACCACTTGAGTCCGGCAGTGAACAAAGAGGCCTGGACAGAGGAGGAAGACGAACTC
ATCTTCACGACACGCGACCGCATCGGCAATCGCTGGGCCGAGATCGCTCGTTTGCTGCCTGGACGGACGGACAACGCGAT
CAAGAACCGCTACTACTCCACCATGAGACGCCAGGGGCGTCAGCTCCGTGGCAACAAGAGCAGATCCCCGGACTCAATGT
CCGAGACCTCTGTGACCGATGACAAAGCCGGCGTCATGTCAGGATCCTTAGTGTCCTCGGAGACAAGCGCGGCTGAACTG
CCCATGGAAAACATT
```

>PITG_08807
ATGCAGATCTCGGCCATGCCCATGACCGCTGTAGACTCCATAATTTTGTCGTCGTCGGACTCGTCAATCGTTAGTATCAC
CAGCAACGACGGTGTGCAAGATAAGCGTCGCCCGTGGACACCCGAAGACGATGCCGTTATCCTACGCTTCGTGCACGAAT
GTGGCACTAAGCGCTGGGCGAAGATTGCCTCGCTGCTACCCGGTCGTACCCCCAAGCAGTGCCGCACACGCTGGCTTAAC
TACCTGGACCCCAACATCGACAAGGCCCCTTGGCGCGCGGACGAGACGCAGCTCATCCTGGCGGCACAAGAGCGAATGGG
CAATCGCTGGGCTGAGATCGCTAAGCTGCTACCTGGTCGGACCGACAATGCCATCAAGAATCACTGGTACTCGACGTATC
GCCGTCGCTGTCGCCAAGCGGCAAAGCTACAAGAGAAGGTGAATCAGTATCAAAACCGAATGGAAATGATAACCTCGGAG
CAAATGATCATGGCGACAACAATGGCAATGCCCAAGCAACACCAGGGCCAGCTGGCAACAGAGGCAACTTCAATTTGGAA
CGGGGCAATAGCAGCTTCCGCTGCATTAGCTTTGCCGTCCCCGCTGTTAGTAAGCTCGCCACTGGGAGCCGGATGCTTCG
GATCGGGTATTCCGACGTTGCTGTCACCATTGCGATTATCACATAAATCCACTTCGTCTGTGGCCGTGTCAATGGCAATG
CCCTTGCCGATGCCCATGACACTACCATCGCCAAAGGCTCTTTTTCCCACAGAGAATAGCGCTTTGCCGTCGTTTTTCAC
TCCTCAGTTTACGAGCACGGTGACACTGAACGCAAATGTGCCGCACAAACGACCAACGTTACAAAACCACCAATCGGCGT
GGATGGGTCTCCCCGGACTGCAATTAACGGGCAACGAATTTTTATCGTCAGGCAGTAGCTGTGGGAGCATGTCGAGTGCA
GTAGATACGATCCCTGCATCGGAGATCGACAAGACGCGGTGGATGAACAAGTCGAGAGTGGAGGTTCTGAAACGACAGGG
CACTCCTGGACGTGAACGCAGCAACTCGGCTGATTTATTTCTCGACTGTGTTGAGATGATGAATCCTATAACGGCAACAC
TGAACAAATGCAACTCGGCAATGAGTATTGCAACAAATGAAACCGACGATGAAGGTAGCGACCAACAGTCCTTGGCCTCT
CCGATGGACAGCGAAAATCGAAACAACTGCAACTTTCCTGGCGTGCAAAAGCAGTGGTGGGATCACATCGATATGGGCTT
CGGTGCCAAGAACAACGGGGGCTTCTGCCCTAGACAAAACCAGTTTCAAACGTGA
>PITG_05990
ATGGTTGCCGAGTCAGCAGTGAAGCGCGGTCGGGACGCTGTGAATGCCGAGCCACACGCAGAACAAGCGGGCGAGGCCCA
GAAGAAGAAGAACGGCGGCGTCGTGAGCAAGCGCTGGACGCCCGAGCAGGACGAGGCGCTTAAGAAGGCCGTGGCCGAGG
TCGGGCACCGCAACTGGATGGCTGTGGCCGAACGCGTGCCAGGACGTGACAACGCGCAGTGTTTACAGCGTTGGAACAAG
GTTCTAAAGCCTGGATTAGTCAAAGGTCCCTGGTCAGTTGAGGAGGACGCGATGCTCATGGAGATGATGCTTAAGGGCTA
CGACAACTGGCGCCAGGTGTCAAACAGCATTCCCGGACGAACTGCAAAACAGTGCCGCGAGCGTTGGCGAAACCGATTGG
ACCCAAGTATCAACAAATCACCATTTACCGAGGAAGAAGACGAAGCCATCCAGCAAGCGTACGAGAAATATGGCAACCGC
TGGACGCAGATAGCAGAGCTTCTACCAGGACGTACAGAAGACGCTGTAAAACTCCGATGGAAGGCGCTGAACCCGAACCA
GAAGACATATACAAAGCTTGGCCGTCCGCGTCTTCTAAACTCGGATGACGCGCCGCCAGTGTCCACGTCGCCAGTCAGAC
CCAATGTGCCATATACCGCCCCAGCACAACCTGTGAAGATGGAAATGTCGCATCACCCAGCTCCTGTGAACAACAATGCT
ATCAGCAATACGACTCAAGTAAATCTGGAGCCTATTTTGACGCCTCTGGAAGACGAGACGGACGAAAAAATGAGCGCGGA
GGACGTCATGATCCTGAACGAGTTTTTGCGTGGCCACTCGAACAATTCGCTCGGGAACGGTAGTTTCAAGAGTTTGCTAT
CTGCAACCAGTTTGCGTGACTTGGGCGATGATATCGACGCTGCGATTATGCGGATGGAGTCGCAGTCAGGGGCGCACCCG
CTGACGGAGTCTACAGTGGAAGACTCGACGGCGTCCAAGCCTCTTCCACTGGGCCGTCCTCCGCACCGAGTGCTTAATAA
GATGCTGAGTCTTGGAGACACAAAGAGCGCGAGTTTGCGCTCGATTACGGCAGATGTGGAGCCCGACGATACGTGGATCA
ACGGAGGCTTGACGCGACGTCTCAGCAGCTTGTCGGTAAACGGAGAAGGTCTTTTCGAGCAGGAACTTGTCGGTGGCAAC
CAGAAATCGAGCTCTATGGACTTGTTCGAGCAGCAACTGGCGTCACTTCATGATGCAGGCTCCCTGATGAGTCTTCCGTT
GGATTTGGGCGATGAAAGCAAGTCAGATAGCGCAGTGGCGGCGGCTGCATACGATGCAGTGAATGCTCCTCCTGCCACTT
CACATGGCTCTTTGCAGCGCTTGATCTTGGAGGATGAGATCCCAGTGCACGGTCTATCGAACCAGACAGCGACGACGGAC
AAGAAGACGATGACAGCACAAGATTTCTACAGTAGCGCCATCCCACACGACCAACTGTCGACAGCACACCGCTTTGAGAT
CTAG
>PITG_05989
ATGTTGCATTACAGCAAACGCGAGCGCGAGGCAACAGACGATGCGTTGGGTTCGCTCCATGAAACTCCGAGCGAAGCGCA
AAAGCCCCGCGAGGATGAGAAGGGCAAGCGCTGGACCACTGACCAGGACGATGCGTTGCGCAAGGCTGTAGACGAGTTCG
GCCAGCGCAACTGGAAGGCCATCGCCTCGCGTGTGGATGGACGCAACCACGCGCAATGTTTGCAGCGCTGGAACAAAGTG
CTCAAGCCTGGACTGGTGAAGGGCCACTGGTCTTTCGAGGAAGACAGCACACTGGAGCAGATGGTGTTGCAAGGCTGCCA
CTCATGGGGTGAAGTGGCCGCTCATATCCCCGGAAGAACAGCGAAGCAGTGTCGCGAGCGATGGAGGAACCACTTGGACC
CGAGCATCAACAAATCACCTTTTACACCTGAGGAAGACACAATTATCCAGGAAGGGTTCGAGAAAATGGGCAACCGATGG
ACACAAATTGCTGAATTGTTGCCTGGACGTACGGAAGATGCGATCAAGTTGAGATGGAAAGCATTGAACCCGAACCAAAA
GGTGAAAGCCAAGCCTGGTCGACCGAAACTCATGCCAGGAATGACAGTCAACAAGCAACGCTCGATGGCTCCCCCCACAC
CTGACGATGTTGCGGCCACTCTGATGAATGGCCCAATTACCATGCCAGAGTTGCCCTCTTACGGAAATGGGTACCCTGAC
CCAATGCCCTATCTCGACAATAGTGTACCCATTACACCAATGACAATGCCACCTCAAATGGCAATGCCTCACGAAGAGTC
TCTCCCTACAACACAAGTACCCATGCCCAGCCAATACCCAGAACCTATCGTTGAACCTCTGAGTGATGAGGTGAAGGCCG
AAAATGACATAAATGATGCAGCCATTCTCAAGGAATTACTACGCAGTCACTCGAACAGCTTACTGAGCTTTGGTAGTGCA
CGCGGCTTGACCTCATTTACAGACATGTCGCCCGAAGACTTGCTTGCAAGTGGCGAACTGGACGAAATGTTCCGTGCAAC
AGTGTCAATATCCAAGGAAAGAGGCGAGAGGGGGCGTCTCTCAAGTAGCAGTATGATGGACAGTTTGACGAGCTCCTTTA
AGAACCCAGAGTCGTTGCAGAAGGCTGTACAGAACCTGGATCACGAAGACAAGCATCTGTTTCAAGGACTTATTGACAGT
TGGCGGGCTCAAAAATCGACTGGTGATGCAAATATCCACGAGGACCATATTGCCAATTTGCCAGTGGAGACGAACGCGTA
CTCGCAACATAGTTTCGAAACGAGTAGTGGCCACAATTTGACGCATTCACTGGATTGTGAGCAGACACGGTCGCTATCTC
GACGAAACTCGAACGGCATCTCGTACGAACTTGGCAACATTTCGAATGATATTGACGATCTATTGGACTCGGATCTCATG
CGCCCGATTCGTAAGGGCAAGATGTGCTAG
>PITG_00038
ATGGCGACAGTAGAAGACTCGCCTGTAAGCTCTGCCAAGCTGACGGAGCTGGGTAAGGCTCTGGACCAGACGTCTATATC
CAATGTCAATGGTAATATATCGCCCGCCGCTTCGCACGAGGCGCCCAGCAGTGAGGTGGACATGGACGCCACCGAAATGC
CTAGCACTCCTGCACCCAAACTGCCAGCCAAGAGCCCCAAGAAGCGCGGGCGTACCGATAGTTTAAAGAAGATGCTGCCA
GCACCAGCTTCGCAGCGTCATAAGCAAGTGACGAGCTCTCCACAGTCTTCAATGCAAGGAAGCAGCGGCACGGAAGAGGA
ACCGTCAGCGTCGCCGGCCCCGTCGTGTGACACTCCACCACAGGCACAGTCGCAGCAGCGTCCACACACTCAGCCAGCGA

AATTGATGGTGAAAGATCGGAGAGGCAGCGGCATGGGCGTCCCACGAGCGCATTTGATTCTCAACGCAGTGAAGCAGGCG
CCGCCAGCGAAGGAAAAATCGAATCCGAGACGGTGGTCGAAGCACGAGGACGAGTCGCTGAGGTTGGCGGTCGAGCGGAG
TGGCGAGCGCAACTGGAAAGCCATTGCCGACCAGGTGCCTGGACGGAATCACACGCAGTGTCTGCAGAGGTGGACGAAAG
TGCTCAAGCCAGGCTTGATCAAGGGCCACTGGACCCCGGAAGAAGACGGTAAACTGCGGGAACTGGTGGCGGAAGGCAAA
AAGAACTGGGGACAAGTAGCCTCGCTCATTCCTGGACGAACATCCAAGCAATGTCGAGAGCGTTGGTGTAATCATCTGGA
CCCCAACATTAACAAGggATCTTACACGGAGGATGAAGACAAGATTATCGTCGAAATGCAAGCTAAACTAGGCAATCGCT
GGTCCATTATCGCTCAGCAGCTCAAGGGCCGAACCGAAGACGCTGTTAAAATTCGCTGGAAATCATTAATGCGCGGACGA
CGCGCTGCATCCAAAGAAGATAAAACACCTGCAGCAGCTGCTGTGAGCACCGCATCATCTTCTCCTGCCGACGTCGAGCG
GCCCCAAGCTAAACCTGCTAAAGCCAGCCCGGTCCAGCAAAAGAATCGTACCCCCGTCACCACAGCAGAGACAGGCCACA
ACCCGACTGTGAAAAATGAGCGGCAAGTCGCAGCAGAAAGCAAACCGGTGGTCGATGCAGCAGTAGGGAGCATGCCAACT
ACCGTTTTTGTCAAGAATCAATCAATGTCGCACCCAGATATGGCAGCAGCGATGGGAGCCCCCGTGAATTCGCTACACAA
CGTGAGTGACCTGGTGGCCGCGTCAATTCACAACTTCCAGATGAGTCAGCGCTTCCATCCGAACTCGGCGGCGCATCCCG
GCAATGCCAGTTACCCTGGCAATCCGAATCAATTGGTGACGGGTATTGCACCTGGCAACCAGCAACCGCCTGGTGTGTAT
GGCATGGCTGATGGGGGATTTAACGTGAACATCCAGCAACAAATGCTACTACAGCAGCATCCGTCGCAGCATATTCAGCA
CCAGCAAGTACCACCAGCATACCAGTATCCTCAGGGATACACCATGCAGCAGCAAAGTATGGGGGTGATGCCAAACTACG
GAGTGCCCACCAACTTCTCGGCGTCAAACCAAATGCAGATGCAGATGCAGATGCCCATGCCGGTATCATCTTCAATGCAG
CAGTCGATATCGATGCCTCCGACACCTACGTACTCGTCTCAGGCAATGGCGATGGCAATGGCGAACGCACAGTATCAGCA
GCACCAGCAGCATCAGCAGCATCCTCCTCCTGTGCACCAAGGCTTCAGCGGTGCACCGCTTCACCCGCGTGTTGCATCGC
TGGGTACGCCTTCCGGGACGTCGTCTATGATCCTTCCCACAAGTAGCGGTGCCAGCAACACTTCCAATGGCATTGGATCC
AATGCTGGTCTTCCTCCGTCGTCAGCTGCAGCAGCCGCAGGAGGGAAAGCTCTGAACACTCCACGGGAAGAATGGGGTTC
TTCACAGACTCCGCGGTCTCAGATGATTATGACGGAAGGCCACGCATCAGCATACGAGCTGTTCCATCAGCAACGCCTTC
GACTAATGTTACAGGAGCGCGATAAGCAGGGGATGTCACTATCATCGGCTCCATCTCCGGGTCAAGCGCTGCTCACAAAA
GAACTGGAAGCCAACAAGGAGCGACAAGCTCGACAGGCGATCATGCAGAAGGGCTGGAAGAGTGCCGTCGAGTCGATGGT
CTCTTTCAACAGTGTCAGCGATTTATCAACTCTGGACGATCAGCAACGGTTTGATGGCCTACTGGAGAAGGTTTCATTGT
CTGCTTTGGACCCAACAGACGACGAGTTACTGGACCAGACTGTTGACATAATATCAGGA
>PITG_16114
ATGGCGCCGGCACAGCCGCAATCGACCCCCGCCGCCACCGAGGCTGGCGCCGTGACCAAGCTGCAGCGAACTTCCAAGAA
GCGCAAGGCCAGCAGTGACGCGCCCGCCGTCTTCCGAGCCTCCGCCGCTGCGCGTGATGCCAGCGGAGCCTCAGCACGCT
GTTCGCTTTGTGGCTTTAGCGCTTCCACACTGGATCACTTGCTCACTCACGTACGCGAGTCACACGGCCGAGACCACAAG
CAAGCGACGCCCCAGATGGCACTTCCGCTCGAAGTGGCGCAGATGGTGGTGCAGCGGGCGCTGGATCAGGCGGCGCTGGG
AGGCGCCACCAGCGCGTGGCTTGAGGTCATACAGCAGCAATTCAAAGCTGCAGCTGAGATGACGGCTGCACTGGAGCGGA
AAGACGCGCACAGTGTTGCGGTGGCAATCCGCAAGCTGCATGGCCTGACGCAGGCAGGAGATGAGGAGTCAGTGCTAATC
ACATTACAGCAGGCTGCGCATATCTTGCTACAATCGCCACCTCACGCTACGGCAGTCTCGGTGGCGACGATCAAGCTGGA
AAATCAAGCCCATAACCCAGCACAAGCTACAGATAACGCAACGACGTCGAAGCAGAACGGAAAGGATTCTAGTATTCACG
AGGCGGAGGCGGCACAGGCGCTAACGGGACTGCGAGCCACAGCTTCGAGTGCGCAGGAGACGACGACAACTGCGCCCGTC
ATCTCAACGTCGTCCTCGCGTCCACCCGCTGTACCGAAGGCGTCTCCTGCGGCCCCTCAAGCCTCGTTCCCTTCCTTTGT
GCTGGGATCACCTCTGACCATCACAGCGCCGCAGCCTCAAATAGGTAGTTCACATTTCCACCCACCAGAGCTGTTCACGC
CCGGTTCGGTGCGGTCGCCGACCACTCCGTCCTCACTACCAAGTCTCTTTGCGCGCAGGCTGTTGGCCCCGCTGGCGTCT
CCATCCAGTGTAACTGATGGAGACCGTTTGGCGCAGAGTGTGGCTGGGATGGGATACCAAAATCCCATTATCGTCACGGA
CCAACGCAACCCGTTGGGCTCTACTATTCGGCCGGATACTTCCCCAGCATCTCTCACTCCTGTGTTTTATTCGTCATTTC
CGACACTGTCAGCAGCCAATAATGTCGTTAATACTGGCGCTGCTCCTACGAACCGACCGACTTCCGCGTCCCCTGTCAAG
CGCAAAAGTATCACATCCAAACAGTTAAGTGTAAGATGCGATGCCGAAGTTGCTGCCGCTCGTGCTGCTGCATCAACGGC
AACGGCAGAGGCAGCTGCAGACACGTCCGTGCTTGTGGCGTTGGGTCCTATCTCCGTGCCTGGCACACCTAGCACACCTG
CGACTCGCAAAGCTACTCCTCGTCGGTGGACCAAGGAAGAAGATGACGCTCTGCGCGCTGCAGTAGAGAACCACCGCGAA
AAAAACTGGAAGGCTATTGCCGCTCAAGTGCCTGGACGGAACCACACGCAGTGTCTGCAGCGATGGACCAAAGTTCTGGC
CCCAGGTCTCGTGAAGGGTCACTGGTCTCCGCACGAAGATGACCTACTGCGACGCCTTGTGGCGACAGAGCAGAAGAACT
GGGGCGACGTGGCCTCCAAGATCCCAGGCCGCACGTCAAAACAGTGCCGCGAGCGGTGGCACAATCATTTGGATCCGCAG
ATCGTTCGCGGTGCGTACACCCCTGAGGAGGACCGCCTGATCTTGGAGGCACAAGCACGACTGGGCAACCGCTGGTCGGT
CATTGCGGCGATGCTGCCTGGTCGGACCGAAGACGCAGTCAAGATCCGCTGGAAGTCGCACTGCCGTGTGTGGCGAGCGC
GCAAGTACCTGCGTAAGAACCCCAACAGTGATGTGGACATGCTGAGCGAGCCTGCACAGACGCCTCGGACCCCGTTGGTG
CCTCCTACTGGCGACTTTAAGTGGCCGGTGCCGCTTCCAAATGGTCCTGCAGACGAACAAGAATCAAGCAAAACGTGTGC
TGAGGCGACGGCGCAGGCTGTGGGCGGAGAGTAA
>PITG_08697
ATGCGACAGAGACTTACGGTAAATTTTTCTAGACCATCCCCAAAGAGGAGCTTGTCCTGCGCAGGTCTTGACGATCTGGA
AGAGCTGCTTCATAATTCAGATACTACGCGCGCTCCTCCGAACCGTAAACGCCACCACAGCTTAAACAGCGACAACAACT
TCGCTGCGGCTAACTTTGGGACGCCTACATCTCCGTCTCGTCACGATTATCCACCACCTCCTGCGTCCTACCCTGGCGCA
TTGCGTCGTCCTGAGCTTTCCACCTATGGCGAGAGTACGCGCTTCCAACTGGTTTCATCGGCATCCTCGTCTTTCGAGTC
CAGATACACGCAGTCTTCCCCCAACCAGGCGCAATCCATTGATCGTCAGCATTCAGATGGCCAGGACAACGATTATCAGG
ATGACCACGAGTTCGATAATGACTTCAGCTCCAGCTCCGGTGACGATCACGAGTATAAAAGCAGTGGCCACTTCTCCACG
TCAACCTTGACATCGCAGGATGCTTTCGAGCACAGACTGAAACTAGACCATGATGCGCTCACTGGAAAGCGTCTTGATCA
CAGTATTTTCCTCCCCCAGTCTCATCGCACGTCGGGTAGTGAGTGCTCTCCACGCGAAGAGTTTCTCGTCCAGGCGTTTG
GCCCCGGTGGGTTCAAGCTGCATGGTACTTCTGTGTCAAGTAACGATGATTTGGATGACAGTCTTAGTGACGACAGCAGC
AATAACAGGGAACACCTTGTCGCAGGCCATGCCACTGCATTAACAGACGCCGATACAGAGCTCAAGATGTCATTCAACGA
GCTTGAAGCGGATATTCGCGGTGCGATGCATCAGAATCCTCACCATCAGCAACATTCGCCCAGACTGCCAGAGCTTACAG
GGTTATCCCATCAGCCATTCACGCCACCTTCGCATGGCTCGTTCGGACTCAATCGCACTGCCATTTTTCCCCGGAGTAAC

CAGGAAGTACACCACGTCTCAATGTTCGGGCACTCGCAAGATATTCGGCCTCATCTCATTCAAACCCACAAAGAACCAGG
GTTTTTTTCCCGAGAGCAACAGCATCAGAGTCCTTCACGTGTGTCGCTGGCTAAGACGCCACAAAGTAAAGGAACTAGCG
GGGTTCAAACGCCCACCAAATGGCTTCGCGATGAAGACGAACGTCTTCGTGTAGCTGTGGCTCGATTTGGTGGCAAGAAT
TGGAAAATGATCGCCGAGACGCTGGGTAATGGGCGCACCGATGTCCAGTGTCTTCATCGCTGGAACAAGGTGCTAAAACC
TGGTTTGATCAAGGGACCTTGGACACCTGAAGAAGATCGTATCCTGACCAGCCTCATTACACGCTATGGAGTCGGTAAGA
TTCGCTGGTGTGACCTCGCACTGCATTTACCAGGACGGATTGGCAAACAATGTCGCGAACGTTGGTGCAATCATCTCGAC
TCGCGGATTCGAAAAGGTCAGTGGACTCCTGAGGAAGATGATATGGTCTTTCGATGGCAACAGAAGTTAGGAAATAAATG
GAGTGAAATCGCCAAGCTGCTCCCTGGACGCACTGAAAATGCCGTCAAGAATCGTTTCAATTCAGCCGCACGGCGCAAGT
GGCTTATGAATCAAGCCAACAAGTCAGCGGCCACCACAGCAGCGCAGTCAACACCTCCAGCTCCGAACCCCATTCAGGTG
TCTTCGCATGCACAGCTTCAACAGCAACAGACGTTGTTGCAGTCCTCTTCAGCTCACCAAGAGGGCAATGTCTACAACGT
CAGTACGATACCATACGGGAAAGTATCGCCGCTACCCTCGACAGACGCACTGATGGAACATCACCCGCACAATATGATCC
CACCACTTGGACGGCAAAACTTCATGCCGCACTACACACCTCCAACTACCGCGCTATTATCCAGTGGTCCTGTTTTTCGT
CCTGCTGTGGCAATGGGGAGTGAGCAGAGTTTTCAACCGCAAGAACACGACGTATCCACGGAAGTTGGTAAGGGCGACTC
GATGCCGTTGGCGCCACCACCGACGTTCGTGCCTCCCCCCTTGAACTCGCTCTTCCACCCTCCTCCAGTCAATATTTTCT
CAGCTTCTAGCCGCAATTTGCAACATTTAACCAGTCCTGCTAGTGGTGGGGCCTCCCTCACCCCTGTCTTCCCTTTCCCG
TCTCACCTGCCGACTCCAACTCACTCCACCGCACCTGGGGTTATTCTTCCTGCAAAGTCCGACGACAAAGATGTCGACTC
CTCAATTCTCCAAAAGGAAGAAAAACTCGAACGAGATGATTTAGTCCTCCAAGCCGCGTCGGCGTCAAGTTCTGCTCCTG
ATTCCAGTGTTCCCATGGACGACGAAAATATGAACAGCTTCCTTGACAGCGTCGCCCTCGAACTCGACGACATCATGGAA
TAA
>PITG_13133
ATGAGTATCAGCGTCAGTCGAGAGTCTATCTCCAAGCTCATGGGCGAGCTCGTGGACGGTTTCCCCATAGAGGGCGATGG
CCGCTCGTCTATCTCAGACTTCGGCAAGCTGCTCTTCACGGAGGGCCAGGCCAACGGCAGCAGCAACGCGGCCAAGTCCG
GCCAGACAGGGCCGAGTGCGGCCAACAGCAAGCCAACTGCGGCCACCACGAGTCGTGGCACTACTTCTTCGTCAAACGGC
TTGACGACTCAGACTCAGACTGGAAGCAAGCGTGTGTTCACAGAAATTAAGGGCATACCCCGCCAGAAGCAGCATGATAT
TATGAAAACCAAGGACAAGGCCGTGCCTAAGCGCTGGACGCCAGAGGAAGACGACAAGCTCCGTGAGGCTGTGGGCCGCC
ATGGCGAGCGCAACTGGAAGTCGATCGCCGAGGAAGTACCTGGCCGCAACCATACACAGTGTCTACAACGGTGGACAAAG
GTGCTGGCCCCCGGCCTCGTGAAGGGCCACTGGCGGCCGGAAGAAGACGACTTACTAAAAGAGCTTGTGGCCGAGGGCCG
CAAGAACTGGGGCCAAGTGGCCACTCGTATCCCTGGGCGTACGTCCAAACAGTGTCGCGAACGCTGGTACAACCACCTCG
ATCCGAGTATCATTCGTGGCGAATACTCGCCGGAAGAGGACCGCATGATCCTGGACGCACAGGCCAGACTGGGCAACCGC
TGGTCGGCCATCGCCGCCATGCTGCCTGGCCGTACAGAAGACGCTGTGAAGATCCGATGGAAATCGCTCTGCCGAGTGCG
CAAAGGACAAGGCCGTCGTGGTCAACCCGACAAGGGTAAGATGACTCCCAAGGGCGTTATGATGCCTGGCCAGATGATGC
AACAAGGTCCAGGCTTTGACGGTGGAATGGTGAAGAGTGAGGAAGTCGCTGCGTTCTCCATGCACCCACAGCAGCAAGGC
ACGCAAATGGTGCGTCTTCCGAATGGCCAAATGATGGGAGGTAATAATATTCAGAGTGGAGGGCAACACCACTCGATGAT
GGGGAGTATGGGCGGGAACGGGATGATGTACCATAGCGACATGAGTGGCGGTGGGTACGACCCTACCATGGCCCACTACC
GGAAGCCTCCGATGCAGCAACACATTGTGAACAATGTATACGAGCATCCTTTGCCGCCTGCAGGACCAATGGCCGGCAAT
AACTCGCAGGACTATCCCGTTGACCGTCGCTCGAACATGGCCAACATGTCCACTGGGATGTCAGCACATTCACAACATTC
CCAAGCACAGGACGGATACAGACAAGAGTACCAAGGCCCGTCTTCGGGATATCCCTCCAATATGAACAGCGGTATGTACT
CAGCGGGGCCGTCCACGCCTAGCGGAGGGATGAACAACGGACAAATGTATGGCGGCAGCTCGCAGATCAACAATTACCGA
GCTGGAGGAGCCATGTACAGCGACTCACAGGTATCTTCGCATCATTCGCAGCAAATGGCCCATTCGCCACACCCTCAGAT
GGTGTCGTACGACTACGGCATGCCTCCATCGACTAAAAACATGAATGGAGGAGCGTCATCAAGCCACCCGCTGCATCCAG
GCAATAATTCCATGCGAAACCCCGCTTCGGCCTTTGCGCATCGACAGCAGCACTCGCATCAATACGTGGATCGCCCTGTC
CACCTCCCACACCACGTGGCTCACCATGAACAGCAGCACCCGACCATGCAACGCCGTATTCATGCACACGAGCACGACCA
ACCACACCCAGAGGAGGAACCGCCAGTACATCACCAACATCATTTGAAGCCGAGATCAAGTGACATCCAGCCTAAAGAGG
AGCCGCGGCCCGGCCGAATGGCGCAGCCAATGTCCAACCCGGCTGCAATGTTCGCCCGTAGCCAGGCGGCCAAGGCTTCA
CCTTCGGCGGCCAACAGTAATCCAGTCGCGGCCTTCATGCACATGCAACATCATCAGAAGCGTAAAGAACAGCCGAGCGC
GTCTAGCAAGATGGCCCAAGCTGGCCCACCGAAACCTTTCAACCCGGCCGCCGCGTTCGCCCAGCGGTTCCAAGCTGGCC
AGAGACCACCAATGCCCAGTAGCAACTCAGGCTCTACCCGATCTAACGATGATACGGCCGACGAGGATGACGACGGAGAG
AACGGCCGGACTACTGAACCTTCTCTCAAGAAGGTCAAGCCGAGACTTAGCATCGATGCCGCGCGGGCGTCGGCCGCACG
GAGAATGCGCAGCTCCGGTAGCGGCGACAATCTGGCCGGCCGTGGAAGTCTGGATGTGTTTTTGAACGAGATCGGCGACG
TTGGCCGGTTGTCGGACCTCAAGATGGACGGCTTCCAGACGCTAGAAGAGCTGTGGCGAGTCTCAGGCGACATGGATCGA
CTATCGTTA
