# Supplementary material for: Myb Transcription Factors and Light Regulate Sporulation in the Oomycete Phytophthora infestans
Source: PLoS One. 2014 Apr 4;9(4):e92086. doi: 10.1371/journal.pone.0092086 (PMC3976263; doi:10.1371/journal.pone.0092086)
Supplement: Table S2 — Primers used for PCR. (PDF) [file pone.0092086.s004.pdf]

Table S2. Primers used for PCR.

Target	Gene in Broad Institute database	Sequences (5' to 3')
Actin-like promoter	PITG_09284	GCCGTGTACGATAGAAA, TATGTCTGATGCTAC
Transporter RNA	PITG_12808	AAGCTCATGTGCGTGACTGT, CCTCCGAGCAGTACGGAAAC
Avr3A RNA	PITG_14374	GGGACGCCAGCTCACTACATA, TCCGCCAGCTTATCATTACCCA
bZIP RNA	PITG_09190	CTCATTGAGTTTCCTTTTTGAC, TAAGAGCTAGTGGAGTCGATG
CRN2 RNA	PITG_17199, 18497, 18503, 05049	CCGTGCTTTGAAAAACCTGT, CATCTCCAAGTCGGGACCTA
EF1α RNA	PITG_09349	CGGCGGTATTGGCACGGTA, GAGTCCGAAGCGACGAAACCAC
Myb2R1 RNA	PITG_01056	AAGCGCTGGTCGGTCATA, GTGGTTCTTGATGGCATTATCC
MYB2R1 promoter	PITG_01056	GCTCTAATCGATCAATCCACATATCAACGGCTCT, AGGCCATTAAGGGCGCTTGTGTGGGACGTTTAC
Myb2R2 RNA	PITG_08807	CAAATGATCATGGCGACAACA, CCTTTGGCGATGGTAGTGTCA
Myb2R3 RNA and DNA	PITG_06748	TCAACGCTCGAGCAACGGCTG, GCCATCGGTTCGCTGAC
Myb2R4 RNA	PITG_08755	GCGAAATGCTGAGAGTGACGAG, CCCAGGCAGCAGTCGAGATA
Myb2R5 RNA	PITG_00988	GCGCAACTCGGTAGTCCA, GTAAGCGGCGACTGTAT
Myb3R1 RNA and DNA	PITG_16114	CGCGTGGCTTGAGGTCATACA, CCTCCGCCTCGTGAATACTAGA
Myb3R2 RNA	PITG_13133	GTTGACCGTCGCTCGAACAT, GCTGCGAATGATGCGAAGATA
Myb3R3 RNA	PITG_00038	AGGTGCCTGGACGGAATCACA, ATGGACCAGCGATTGCCTAGTT
Myb3R4 RNA	PITG_19851	TCGCTATGTTGATCGCCTAC, GCCAACGACAGCATTAAACGAA
Myb3R6 RNA	PITG_05990	CGTGCGCAGTCAGACCCAATGT, CCGTTCCCGAGCGAATTGTT
NPP1 RNA	PITG_16866	TACCATCCGCCCGAATCCAAC, ATTCTCCAGCGCCGTTCGT
Pks1 RNA	PITG_10884	CGCAGTCCGCTTCTACGC, CCGAACTGCCACAAGTCAACT
Pks1 promoter	PITG_10884	CTTGCTGCAGCGGAAATC, CGCGTCTTCTTTCGACC
RiboS3a RNA	PITG_11766	GATTTCACGGCATGGACTTC, TCTTGGCGTAGGTGGTCTTC
Ste20 intron DNA	PITG_06107	GCTCTAATCGATCAATCCACATATCAACGGCTCT, AGGCCATTAAGGGCGCTTGTGTGGGACGTTTAC
