# Supplementary material for: Myb Transcription Factors and Light Regulate Sporulation in the Oomycete Phytophthora infestans
Source: PLoS One. 2014 Apr 4;9(4):e92086. doi: 10.1371/journal.pone.0092086 (PMC3976263; doi:10.1371/journal.pone.0092086)
Supplement: Table S3 — Numerical values of RT-qPCR data shown in Figs. 3 and 4 . (PDF) [file pone.0092086.s005.pdf]

Table S3. Numerical values of RT-qPCR data shown in Figs. 3 and 4.

Fig. 3A

Hour:	48	60	72	84	96	108	120
Myb2R1	0.02±0.02	0.34±0.05	1.56±0.17	0.83±0.21	1.46±0.12	1.12±0.12	1.69±0.23
Myb2R3	0.01±0.00	0.30±0.04	1.50±0.09	0.66±0.03	1.71±0.13	1.01±0.06	1.83±0.09
Myb2R4	0.03±0.00	0.50±0.04	1.80±0.11	1.55±0.03	1.50±0.22	0.91±0.08	0.78±0.09
Pks1	0.02±0.01	0.02±0.00	0.70±0.10	0.26±0.02	1.98±0.80	1.52±0.16	2.40±0.12
Avr3a	2.53±0.17	2.80±0.10	1.06±0.15	0.55±0.27	0.02±0.02	0.02±0.02	0.02±0.02
NPP1	0.03±0.03	0.29±0.17	0.94±0.36	1.33±0.26	1.36±0.23	1.71±0.11	1.35±0.36
CRN2	0.71±0.19	1.00±0.03	1.00±0.21	0.97±0.13	1.09±0.19	1.28±0.25	0.95±0.62
EF1a	0.90±0.20	0.97±0.21	0.78±0.27	0.84±0.18	0.90±0.20	1.04±0.23	1.57±0.34

Fig. 3B

Hour:	80	84	88	92	96	100	104	108
Myb2R1	0.30±0.16	0.51±0.10	0.56±0.03	1.35±0.11	1.10±0.03	1.18±0.03	1.30±0.22	1.21±0.03
Myb2R3	0.43±0.09	0.42±0.40	0.47±0.47	1.50±0.14	1.10±0.17	1.31±0.28	1.10±0.35	0.90±0.39
Myb2R4	0.50±0.22	1.00±0.06	1.11±0.02	1.32±0.03	1.00±0.13	0.93±0.04	0.82±0.01	0.77±0.02
Pks1	0.12±0.02	0.20±0.02	0.24±0.16	0.76±0.58	1.03±0.38	1.53±0.30	1.27±0.54	2.11±0.66
Avr3a	3.02±0.15	2.08±0.12	2.13±0.12	0.67±0.07	0.25±0.03	0.20±0.07	0.15±0.06	0.15±0.02
NPP1	0.56±0.18	1.31±0.44	0.60±0.00	0.92±0.34	0.64±0.51	1.50±0.17	1.21±0.11	1.39±0.10

Fig. 4A

Hour:	60	72	84	96	108	120
Myb2R1	0.24±0.06	1.08±0.18	0.85±0.03	1.25±0.04	1.12±0.14	1.45±0.09
Myb2R3	0.22±0.04	0.87±0.18	0.59±0.00	1.36±0.01	1.17±0.14	1.80±0.08
Myb2R4	0.52±0.19	1.16±0.12	1.23±0.15	0.99±0.11	1.10±0.23	1.00±0.15
PKS	0.04±0.02	0.28±0.11	0.26±0.01	1.33±0.10	1.86±0.13	2.23±0.06

Fig. 4B

Hour:	60	72	84	96	108	120
Myb2R1	0.06±0.02	0.49±0.11	0.80±0.03	1.04±0.00	1.70±0.10	2.00±0.10
Myb2R3	0.06±0.03	0.35±0.11	0.81±0.06	1.04±0.01	1.62±0.09	2.00±0.07
Myb2R4	0.13±0.02	0.72±0.09	0.98±0.08	0.90±0.03	1.63±0.15	1.40±0.24
PKS1	0.05±0.02	0.09±0.03	0.18±0.03	0.55±0.16	1.59±0.16	2.00±0.32

Fig. 4C

Hour:	60	72	84	96	108	120
Myb2R1	0.10±0.05	0.70±0.11	0.85±0.15	1.05±0.18	1.50±0.23	1.80±0.33
Myb2R3	0.12±0.07	0.56±0.13	0.93±0.20	1.06±0.16	1.43±0.27	1.86±0.30
Myb2R4	0.15±0.07	0.89±0.14	1.13±0.20	0.95±0.14	1.34±0.23	1.40±0.26
PKS1	0.06±0.03	0.09±0.04	0.12±0.03	0.23±0.05	1.28±0.21	2.00±0.31
